# ﻿Revision of *Vanuatubasis* Ober & Staniczek, 2009 (Odonata, Coenagrionidae), with description of seven new species

**DOI:** 10.3897/zookeys.1128.89751

**Published:** 2022-11-09

**Authors:** Natalie A. Saxton, Milen G. Marinov, Seth M. Bybee

**Affiliations:** 1 Research and Collections Division, The Cleveland Museum of Natural History, Cleveland, OH, 44106, USA The Cleveland Museum of Natural History Cleveland United States of America; 2 Department of Biology, Case Western Reserve University, Cleveland, OH, 44106, USA Brigham Young University Provo United States of America; 3 Department of Biology and Monte L. Bean Museum Brigham Young University, Provo, UT 84604, USA Case Western Reserve University Cleveland United States of America; 4 Biosecurity Surveillance and Incursion Investigation Plant Health Team, Ministry for Primary Industries, 14 Sir William Pickering Drive, Christchurch 8053, New Zealand Ministry for Primary Industries Christchurch New Zealand

**Keywords:** Damselflies, *Nesobasis*, South Pacific, taxonomy, Vanuatu

## Abstract

*Vanuatubasis* Ober & Staniczek, 2009 is an endemic genus of damselfly found on the island archipelago of Vanuatu. Previously only three species were assigned to the genus. Here, all known species of *Vanuatubasis* are formally described and treated, including the association of females for known species. The following new congeners are also described: *V.discontinua***sp. nov.**, *V.evelynae***sp. nov.**, *V.insularivorum***sp. nov.**, *V.kapularum***sp. nov.**, *V.nunggoli***sp. nov.**, *V.rhomboides***sp. nov.**, and *V.xanthochroa***sp. nov.** from material collected across six different islands. An illustrated key to both males and females of all species within *Vanuatubasis* is provided as well as distributions for all known species.

## ﻿Introduction

Vanuatu is a small island nation in the South Pacific whose Odonata diversity is largely unknown (Marinov et al. 2019). Prior to this study, the most notable work in the region was performed by the early twentieth century entomologist, L. Evelyn Cheesman, and the 2006 expedition to the northwest coast of the island of Espiritu Santo (SANTO Expedition) (Ober and Staniczek 2009). Cheesman is recognized for her work collecting insects across the South Pacific and specifically in Vanuatu (Touzel and Garner 2018). Among the many species described as a result of her work, are two species, *Vanuatubasismalekulana* (Kimmins, 1936) and *V.bidens* (Kimmins, 1958), formerly classified under the genus *Nesobasis* Selys, 1891. Donnelly (1990) noted that these specimens collected from Vanuatu differed from *Nesobasis* in having characters which may justify the establishment of a new genus: e.g., short cerci and a raised pronotal hind lobe. However, it was not until after the SANTO Expedition when additional specimens were collected, that *Vanuatubasis* Ober & Staniczek, 2009 was formally introduced (Ober and Staniczek 2009).

Several authors have suggested that due to their similarity *Nesobasis* and *Vanuatubasis* are closely related, however, no published phylogeny has yet investigated this relationship (Donnelly 1990; Ober and Staniczek 2009; Dijkstra et al. 2014). Furthermore, the subfamilial placement of *Vanuatubasis* and its hypothesized sister genera have been disputed (see Dijkstra et al. 2014). Donnelly (1990) considered *Melanesobasis* Donnelly, 1984, *Nesobasis*, and what is now *Vanuatubasis* as part of “*Nesobasis* group.” Phylogenetic work, however, has shown that *Melanesobasis* is not closely related to *Nesobasis* as previous authors had suggested (Beatty et al. 2017). De Marmels (2007) excluded *Nesobasis* from the Teinobasinae due to the lack of a cercal spur, noting that this character was different from the basal spine present in many other Coenagrionidae. Following Donnelly (1990)Ober and Staniczek (2009) grouped *Vanuatubasis* with *Nesobasis* and excluded them from the subfamily. In Dijkstra et al. (2014), *Vanuatubasis* was tentatively placed back within Teinobasinae, however, many of the genera in question were not included in Dijktra et al.’s (2014) phylogeny. A more thorough morphological assessment also needs to be done to support their placement here. Thus, the composition of Teinobasinae and the placement of *Vanuatubasis* remains uncertain.

*Vanuatubasis* is just beginning to be understood in terms of diversity and natural history (Marinov et al. 2019; Saxton et al. 2020, 2021). Previous work suggests that *Vanuatubasis* requires alkaline streams and there have been several records of spider-feeding (Marinov et al. 2019; Saxton et al. 2021). Much work remains to be done in the region, as Vanuatu is undersampled compared to other South Pacific countries (Marinov 2015). Here, we formally revise the genus with the treatment of all known species and the formal description of seven new species.

## ﻿Materials and methods

**Figure 1.**
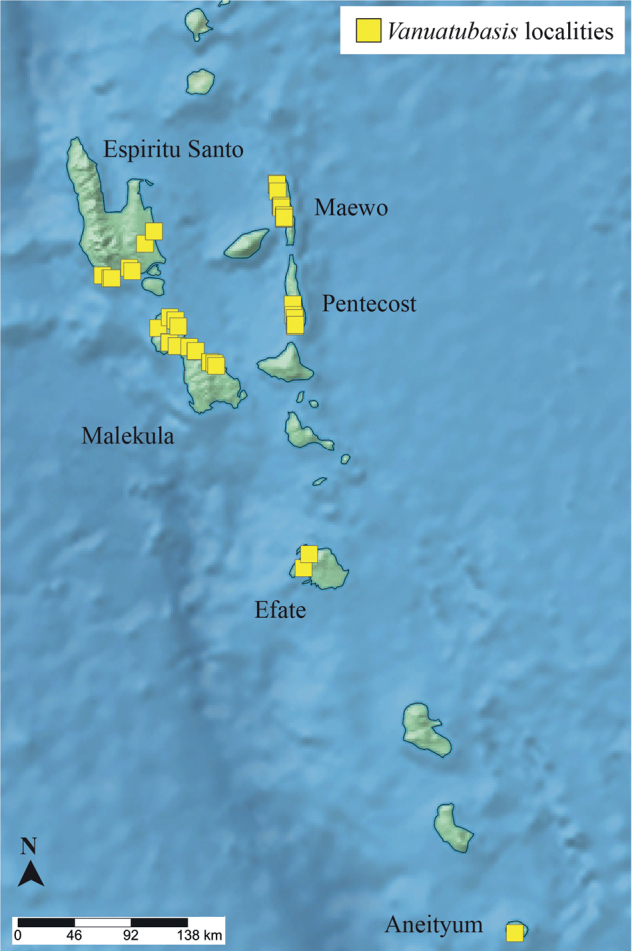
Collecting localities for *Vanuatubasis*. See Saxton et al. (2021) for more details.

A series of expeditions in the country took place from 2017–2019 visiting ten different islands in total (i.e., Aneityum, Tanna, Efate, Erromongo, Malekula, Ambrym, Pentecost, Maewo, Espiritu Santo, and Gaua) (Fig. 1). Specimens were collected using aerial nets and subsequently placed in 95% EtOH. Specimens were examined using an Olympus SZ51 stereo microscope. Images were taken using a Vision Digital passport imaging system and stitched using Zerene v. 1.04 (Zerene Systems LLC, Richland, WA, USA). Scale bars in images represent ~ 0.5 mm.

Terminology employed here mostly follows Garrison et al. (2010) for the general body morphology and Riek and Kukalová-Peck (1984) for wing venation. Three main measurements are given here including full-body length, abdominal length, and hindwing length. Full body length is measured from the labrum to the posterior end of the terminal appendages, while the abdominal length is measured from the anterior edge of the first abdominal segment to the end of the terminal appendages. Abdominal segments are abbreviated in the text to S1–S10, as are hindwing (**HW**) and forewing (**FW**). Nodal indices are given as the number of postnodals in the first row counted from the distal part of the wing/number of antenodals in the first row counted from the distal part of the wing followed by the number of corresponding antenodals in the second row/ the number of corresponding postnodals in the second row. Female specimens were associated and described only for those collected in tandem with males.

Label data for types is given verbatim within quotations with “ | ” to indicate line breaks. Specimens housed in the BYU frozen tissue collection and not in the general collection are indicated as such by the abbreviation “cryo”. All distributions given are in the country of Vanuatu. The following abbreviations for institutions are used throughout the text:

**BPBM**Bernice P. Bishop Museum, Honolulu, Hawaii, USA;

**BYU** Monte L. Bean Museum, Brigham Young University, Provo, Utah, USA;

**MNHN**Muséum National d’Histoire Naturelle, Paris, FR;

**NHM**Natural History Museum, London, UK;

**NZAC**New Zealand Arthropod Collection, Auckland, NZ;

**SMNS**Staatliches Museum für Naturkunde, Stuttgart, DE.

## ﻿Results

### ﻿Order Odonata Fabricius, 1793

**Suborder Zygoptera Selys, 1854**

#### Family Coenagrionidae Kirby, 1890

##### ﻿Genus Vanuatubasis

Taxon classificationAnimaliaOdonataCoenagrionidae

Ober & Staniczek, 2009

40731BA1-2854-534B-B1A5-C26BC55B91B4

###### Type species.

*Nesobasismalekulana* Kimmins, 1936 (by original designation).

###### Diagnosis

**(adapted from Ober & Staniczek, 2009).***Vanuatubasis* resembles *Nesobasis* but can be distinguished by the following characters: cerci of males broad and short, always shorter than the paraprocts, paraprocts forceps-like, apically curved inwards (continuously curved medially), each ending with a dark tip; protonal hind lobe raised and medially protruding to obtuse or acute apex, ventral lobe expanding dorsally, and leveling up approximately with the dorsal carina.

###### Etymology

**(feminine).** The name of the genus is derived from its distribution within Vanuatu, and the Latin suffix -*basis* which means base or foundation (see Ober and Staniczek 2009).

##### ﻿ Vanuatubasis bidens

Taxon classificationAnimaliaOdonataCoenagrionidae

(Kimmins, 1958)

499F80BF-B68B-555B-A90D-A54C21FF98D7

Figs 2 , 20A , 21A

Nesobasis bidens Kimmins, 1958: 239–241 (species description); Ober and Staniczek 2009: 490–492; Marinov et al. 2019: 14.

###### Material examined.

***Holotype*. (1**♂ **NHM)** “Type” “NEW HEBRIDES:| Aneityum. | Red Crest: 1,200ft. [sic] | 3m.N.E.of Anelcauhat. | vi .1955.” “L.E.Cheesman. | B.M.1955–217.” “Nesobasis | ♂ bidens Kim | D.E.Kimmins det. 1957 | TYPE.”.

###### Additional material.

**(3**♂♂, **3**♀♀ **BYU)** “Vanuatu: Aneityum: | Anijemhag River, 12.v.2017 | 20.2180°S 169.8012°E, coll. | S.M.Bybee, M.Marinov” *Vanuatubasisbidens* was known by males only. Here we describe the female.

###### Description of female.

***Head***: Labium overall pale beige; labrum pale green, darkening posteriorly, with dark brown postero-lateral edges and medially, a black spot at posterior edge; anteclypeus, genae, and mandibles (except for reddish tips) greenish yellow; postclypeus greenish yellow medially, with a black bar that begins medially and extends to the anterior edge, not extending to anterior corners; frons yellow, abruptly changing to black posteriorly; scapes and pedicels black, flagella dark brown and lightening apically; vertex and rear of head black, with bronze shimmer and white pruinescence; three pale ocelli with beige patch apical of the median ocellus; eyes cream-colored, although likely different color in life.

**Figure 2.**
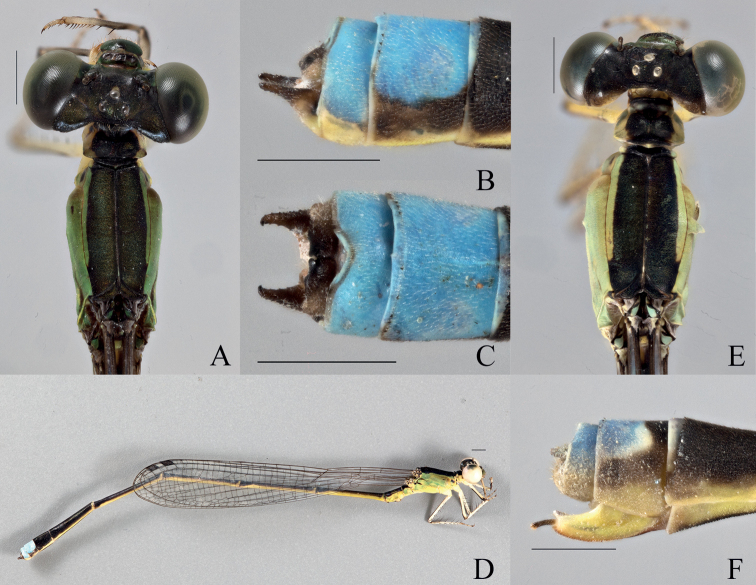
*Vanuatubasisbidens* (♂ BYU) **A** dorsal thorax **B** lateral terminalia **C** dorsal terminalia **D** lateral habitus *V.bidens* (♀ BYU) **E** dorsal thorax **F** lateral terminalia. Scale bars: 0.5 mm.

***Thorax***: Prothorax dorsally black with bronze shimmer; laterally yellowish green; pronotum black medially with greenish yellow edges, postero-lateral corners rounded to obtuse angles and weakly explanate, hind lobe raised and slightly curved outward medially, extending to point that protrudes posteriorly; mesostigmal plate black with green lateral edges, roughly triangular and staying approximately level across the outer surface. Pterothorax with black carina; laterally, mesepisternum with black stripe reaching the dorsal carina and reaching the mesopleural suture posteriorly across ~ 0.5 mm, but only reaching ~ 2/3 of the mesepisternum on the anterior and medial portion; yellow stripe located on anterior 1/3 of mesepisternum, not quite reaching mesinfraepisternum, and extending just past the mesopleural suture; mesepimeron overall pale green with yellow extending down from mesepisternum, short, dark brown line on posterior end of interpleural suture; metepisternum overall pale green with short, black line located on metapleural suture near the base of the wings, extending ~ 1/6 the suture’s length; mesinfraepisternum yellow-green with small, dark-brown spot located medially; metepimeron pale green and turning beige dorsally; coxae, trochanters, and femora dorsally pale brown and ventrally pale beige with black spines; tibiae pale brown with slightly darker, and smaller, spines than that of the femora; tarsi beige with dark brown edges and smaller spines; pale brown tarsal claws that darken apically to reddish tips, claws with a small tooth located on the basal 1/4 of their length.

**Figure 4.**
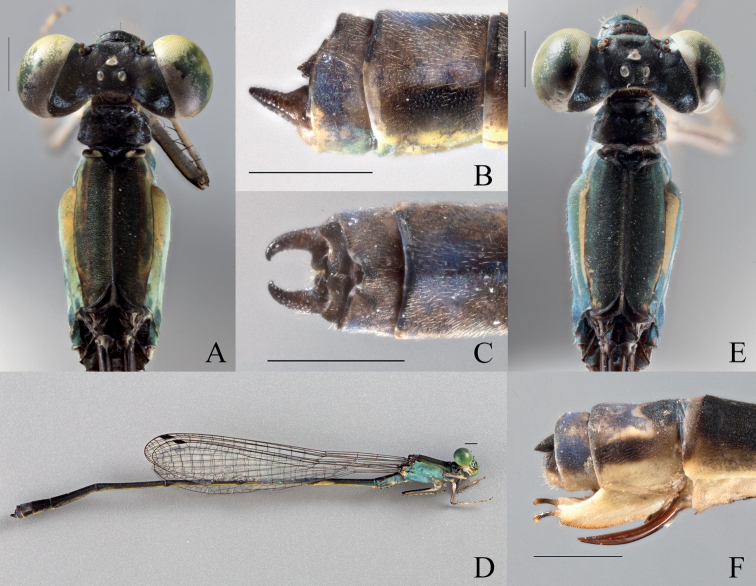
*Vanuatubasisevelynae* Holotype (♂ BYU) **A** dorsal thorax **B** lateral terminalia **C** dorsal terminalia **D** lateral habitus *V.evelynae* Allotype (♀ BYU) **E** dorsal thorax **F** lateral terminalia. Scale bars: 0.5 mm.

***Wings***: Hyaline; venation dark brown; pterostigma elongated rhomboidal dark brown and lightening towards the edges; CuP approximately halfway between antenodals in all wings; arculus originates slightly distal of second antenodal crossvein in all wings; discoidal cells unequal with FW dorsal edge being 1/2 as long as HW. Nodal index: 14/2–2/13 in FW and 13/2–2/12 in HW.

***Abdomen***: Overall, yellow with black dorsal stripe, that lightens laterally, extending from S1–9, dark brown lines encircling the posterior end of S1–S5, and pale brown setae; S1 with anterior 1/2 beige and latter 1/2 pale green; S2 pale green; S3–S8 laterally yellow; S9 yellow, with dorsal stripe extending ¾ of lateral view posteriorly; S10 laterally blue with brown edges, dorsally with blue patch extending ¾ of its length. Ovipositor overall pale yellow and reddish brown ventrally; stylus rounded, dark brown and lightening apically; gonapophysis reddish brown. Cerci roughly triangular, brown, and narrowing to a slightly rounded apex.

***Measurements* (mm)**: total length 35–36 mm, abdomen 29–30 mm, HW 21–22 mm (*n* = 3).

###### Diagnosis.

**Male.***Vanuatubasisbidens* can be distinguished from other known species of *Vanuatubasis* by a black pterostigma, bright blue abdominal S9 and S10, relatively straight black cerci that only curve medially on their apical 1/3, the presence of cercal teeth (although difficult to see in some specimens), and by having the lateral lobes of the genital ligula covering the sclerotized portion of the first genital segment. **Female.***Vanuatubasisbidens* can be distinguished from other females in this genus by green thoracic coloring, and pale colored cerci surpassing the length of the stylus.

###### Variation.

**Male.** The sinusoidal shape of the cerci is not as pronounced in some individuals nor are the cerci “teeth” as prominent. Color varies from yellow to green, likely due to the maturity of the specimen. **Female.** Variation in color due to maturity of specimens, yellow immatures and green mature. Terminal ends of cerci are sometimes more pointed than that of the description above.

###### Distribution.

Aneityum, Vanuatu.

###### Notes.

This species was previously only known from one male. Here, we expand the number of known males collected as well as confidently associate the female. Kimmins (1958) noted that the holotype of this species, described as having yellow thoracic coloring, was likely an immature specimen but only had a single specimen and could not confirm this hypothesis. Additional collection efforts have confirmed Kimmins’ hypothesis and found that the mature individuals are green and immature individuals are yellow (see Marinov et al. 2019: fig. 9).

##### ﻿ Vanuatubasis discontinua sp. nov.

Taxon classificationAnimaliaOdonataCoenagrionidae

9FBE540A-C4F3-5AE2-9A76-4099061EF5D9

https://zoobank.org/CF66CBD3-F145-46C1-8A7D-549189814285

Figs 3 , 21B

###### Type material.

***Holotype* (1**♀ **NZAC).** (NZAC04230977, New Zealand Arthropod Collection, Auckland, New Zealand), locality data: Malekula Island, Litslits (-16.1460, 167.4653; 45 m a.s.l.): 24 May 2018; S. Bybee & G. Powell leg.

***Paratypes* (5**♀♀ **NZAC).** Malekula Island: 1♀ (NZAC04231068), Losinwei (-16.1079, 167.3273, 29 m a.s.l.), 06 May 2019; 3♀♀ (NZAC04231069-71), Brenwe (-16.083, 167.275; 180 m a.s.l.), 22 May 2019; 1♀ (NZAC04231072), Wiaru River (-16.0787, 167.2726; 188 m a.s.l.), 13 May 2019); all S. Bybee & G. Powell leg.

###### Additional material

**(1**♀ **NZAC).** Malekula Island, Stretch of Lakatchkach River flowing through Postanle Area (-16.1437, 167.4671 to -16.1474, 167.4649; 15–51 m a.s.l.): 17 May 2017; M. Marinov & S. Bybee leg.

###### Description of holotype.

***Head***: Labium pale yellow; labrum, mandibles (except the reddish tips), clypeus and frons up to the midline between antennal bases and genae along the eyes up to dorsal level of scapes yellow with darker spots as follows: dull fulvous spot (to pale brown) along the posterior edge of the labrum; on postclypeus three areas with darker centers and diffused edges which connect to each other and with a transverse bar at the middle dorsal surface forming a T-shaped structure and dark fuscous to dark reddish at the dorsal corners of the mandibles at the ends of the lateral proximal corners of the postclypeus plus lateral sides of labrum running down for almost the whole length to the anterior edge; scapes yellow, pedicels pale brownish, flagella missing, scape: pedicel 0.3; vertex black with slight dark red sheen with a dull yellow spot in front of the median ocellus; rear part of the head yellow continuing up to occipital area and visible from the dorsum, yellow almost fully interrupted dorsally on the occipital bar between the postocular lobes except a small elongated spot in the middle area.

**Figure 3.**
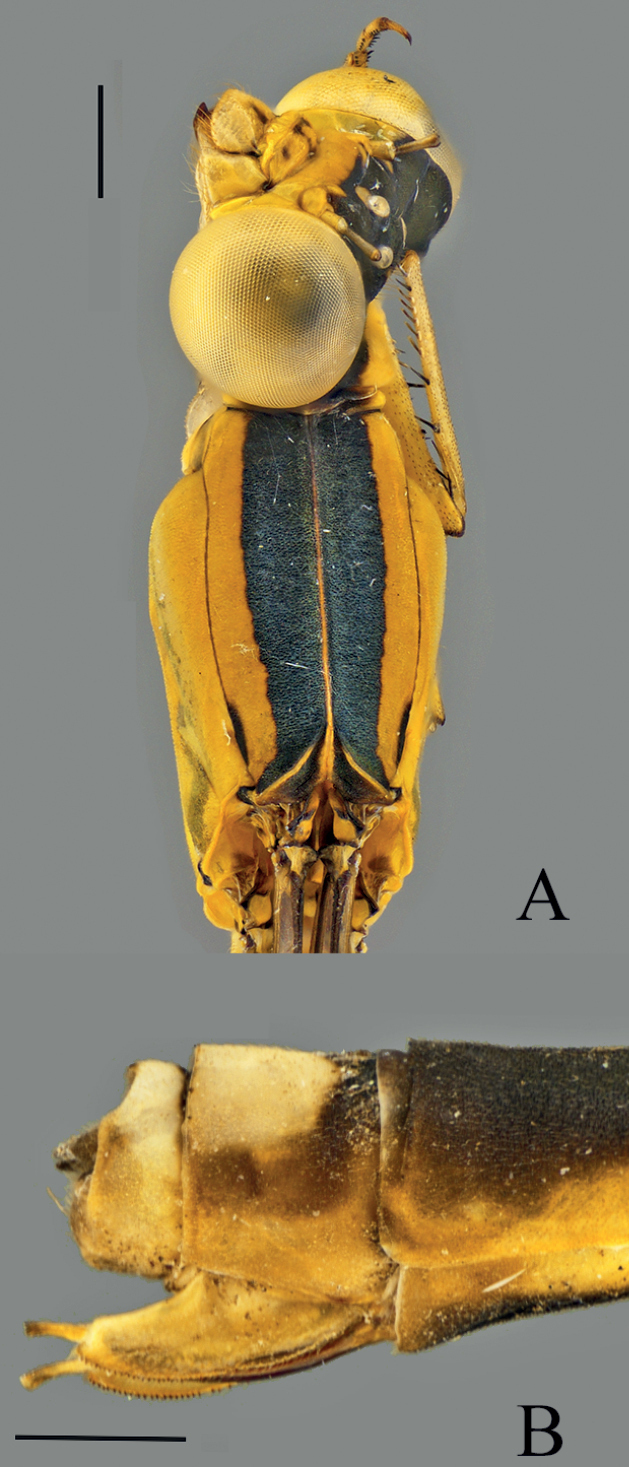
*Vanuatubasisdiscontinua* Holotype (♀ NZAC) **A** dorsal thorax **B** lateral terminalia. Scale bars: 0.5 mm.

***Thorax***: Dark wide parallel sided bar with slight reddish sheen passing on the dorsal part of pro- and pterothorax encompassing ¾ of the dorsal part of the mesostigmal plate and 2/3 of mesepisternum with a faint light line on the posterior edge of the middle prothoracic lobe and dorsal carina, at the posterior ends the dark area curves ventrally towards the mesopleural suture with its edges making two consecutive semicircles joining the suture at its very posterior ends; dark elongated spots with same color developed also as follows: one oblique occupying the upper part of the mesinfraepisternum, two sitting on the dorsal edges of the meso- and metapleural sutures at their posterior ends and faintly connecting to two dark shiny spots at the alar areas by a tiny bridge with the same color; legs almost entirely yellow saved for the faint barred dorsal area on all femora (very weakly developed to almost invisible on the front legs and darken like a dashed line towards the hind legs), dark spots at the joints between femora and tibiae and all the leg spines, claws orange with darker reddish tips; mesostigmal plate roughly triangular shape with tip wide rounded to almost straight; posterior edge of posterior edge of the prothorax dorsal edge interrupted at the middle.

***Wings***: Hyaline; venation generally dark especially at the distal ends becoming lighter towards the bases with pale spots at the nodus at the outer sides; pterostigma elongated rhomboidal light fulvous to almost transparent; CuP approximately halfway between antenodals in all wings situated very closely to the wing petiolation almost at the point where CuP ad AA is leaving the wing edge; arculus originates at the second antenodals in front wings and slightly distal to it in hind wings; discoidal cells dissimilar in shape – in front wings anterior side is ~ 1/3 of the posterior and in hind wings anterior side is slightly longer than 1/2 of posterior; nodal index: 12/2–2/12 in front wings and 10/2–2/11 in hind wings.

***Abdomen***: Almost uniformly dark fulvous on the dorsum from S1 to S9 with yellow developed as follows: isosceles triangular spot on at the base of S1, all segment from S3 to S7 with lines at the base coming from the ventral and continuing up to almost meeting at the dorsum with yellow fainting in intensity towards the posterior segments; dark on S9 occupies a little more than 1/3 on the dorsal part, descends laterally and ends at the posterior end of the segment as a very faint dark area, rest of S9 beige on the dorsum; S10 dark base as deep reddish line overarching the dorsum and descending towards the venter, rest of dorsum beige; ventral side of the abdomen yellow except for: narrow dark lines running on almost entire length of the ridges of the sternites 2 to 8 (slightly to widely expanded on second and eight segments respectively); cerci pale brownish; posterior end of sternite 8 ending in a very low blunt tooth; ovipositor orange yellow aligned with the posterior end of S10 and styli surpassing the posterior ends of the cerci by 1/5 of their length.

***Measurements***: total length 38.5, abdomen 32.0, hind wings 23.5.

###### Diagnosis.

**Female.***Vanuatubasisdiscontinua* is characterized with pale yellow ground color with dark markings on the thorax and dorsal part of the abdomen. It can be distinguished from other congeners where females exhibit similar type of coloration at various stages of their life (e.g., *V.bidens*, *V.rhomboides* sp. nov.) by the following combination of characters: long pedicel (scape: pedicel < 0.5), maculation on postclypeus present and brown cerci.

###### Variation.

**Female.** Greatest variations observed on the shape of the markings on the postclypeus where the T-shaped structure could be reduced to two isolated spots. Dorsal carina along the posterior edge of prothorax not interrupted in two specimens. Dark elongated spots on the mesinfraepisternum diffused in most specimens and missing in one paratype. Two paratypes with pale markings on the dorsum of S9 close to the posterior end of the segment. Ovipositor surpassing S10 in one specimen.

***Measurements* (mm)**: total length 35.0–38.0, abdomen 29.5–31.5, HW length 21–23 (*n* = 5).

###### Distribution.

Malekula, Vanuatu.

###### Etymology.

The specific epithet for this species is derived from the modern Latin word *discontinuus*, –*a*, –*um* = discontinuous, referring to the interrupted dorsal carina on the prothorax (declinable adjective).

###### Notes.

This species is only known from female specimens as no males were able to be associated. One female was excluded from the type material as it generally resembles the paratypes, but has the following differences: (1) posterior edge of the middle lobe of prothorax with incision having bilobed shape, (2) elongated spot on mesinfraepisternum darker than other paratypes, (3) dorsum of S10 with a diffused longitudinal bar joining the posterior end of the segment enclosing two pale spots, (4) smaller size than paratypes: total length 30.5, abdomen 25.2, HW length 19.5.

##### ﻿ Vanuatubasis evelynae sp. nov.

Taxon classificationAnimaliaOdonataCoenagrionidae

1CE81D42-0BD9-534D-AEE8-C965351459BE

https://zoobank.org/5974BE63-DD97-411B-998E-6BD01F582838

Figs 4 , 20B , 21C

###### Type material.

***Holotype* (1**♂ **BYU).** “VANUATU: Santo Is: | Coulons, -15.2957 | 167.1616092, 28 v. 2018, | coll. S. Bybee and G. Powell’’.

***Paratypes* (1**♂ **1**♀ **BPBM, 7**♂♂ **BYU, 3**♂♂ **NHM, 3**♂♂ **NZAC). (1**♂ **1**♀ **BPBM, 3**♂♂ **BYU, 3**♂♂ **NHM, 3**♂♂ **NZAC)** Same label data as holotype. **(1**♂ **BYU)** “VANUATU: Santo Is., | Coulons, May 20, 2019 | -15. 2955, 167.1614 | coll. SM. Bybee, GS. Powell | #BYU-VU-2019’’ “OD1710” **(1**♂ **BYU cryo)** “VANUATU: Santo Is: | Pelmol, -15.6078, 166.7849, | 4.vi.2018, |coll. S. Bybee and | G. Powell” “OD1709” **(1**♂ **BYU cryo)** same data as holotype; “OD1710” **(1**♂ **BYU cryo)** “VANUATU: Santo Is: | Coulons, -15.2957 | 167.1616092, 28 v. 2018, | coll. S. Bybee and G. Powell’’ “OD1711”.

***Allotype* (1**♀ **BYU).** same label data as holotype.

###### Description of holotype.

***Head***: Labium overall pale beige; labrum blue-green, darkening posteriorly, with dark brown postero-lateral edges and medially, a black spot at posterior edge; anteclypeus dark green; genae, and mandibles (expect for reddish brown tips) green with paler anterolateral edges; postclypeus overall covered by a black bar that begins medially and extends to the anterior edge, not extending to the dark green, lateral corners; frons dark green, with medial black and extending posteriorly; scapes, and pedicels black, flagella dark brown and lightening apically; vertex and rear of head black with line of setae, a bronze shimmer, and white pruinescence; a pair of white post-ocular spots present; three pale ocelli with a yellow patch apical of the median ocellus; line of setae behind vertex; eyes yellowish green.

***Thorax***: Prothorax dorsally dark green to black with a bronze shimmer; laterally blue and green; pronotum black, latero-posterior corners rounded to obtuse angles and explanate, mid-line obviously indented across the pronotum, hind lobe with raised ridge that is shorter than the width of the pronotum, is curved outward medially, and extends to a sharp point that protrudes posteriorly; mesostigmal plate dark brown with yellow-green edges, interior edges raised to form protruding lobes. Pterothorax with black carina; laterally, mesepisternum with black stripe reaching the dorsal carina and extending to the mesopleural suture posteriorly across ~0.5 mm, but only reaching ~ 2/3 of the mesepisternum on the anterior and medial portion; green stripe located on latter 1/3 of mesepisternum and extending past the dark brown mesopleural suture; mesepimeron overall blue with green extending down from mesepisternum, metepisternum overall blue with short, brown line located on metapleural suture near the base of the wings, extending ~ 1/6 the suture’s length; mesinfraepisternum bluish green with dark brown spot located medially; metepimeron pale blue with white pruinescence; coxae, trochanters, and femora dorsally brown to black and ventrally pale beige with black spines; tibiae pale brown with smaller spines than that of the femora; tarsi beige with dark brown edges and small, dense spines; pale brown tarsal claws that darken apically to reddish tips, claws with a small tooth located on the basal 1/4 of their length.

***Wings***: Hyaline; venation dark brown and thickening towards the dorsal edge; pterostigma dark brown and rhombus-shaped, with edges being the darkest; CuP slightly distal than halfway between antenodals in HW; arculus originates just distal of the second antenodal crossvein in HW and slightly proximal to it in HW; discoidal cells unequal with FW dorsal edge being 1/2 as long as HW. Nodal index: 12/2–2/12 in FW and 11/2–2/10 in HW.

***Abdomen***: Overall yellowish green with black dorsal stripe extending from S1–S9 and lightening to brown laterally, brown reaching ventrally at posterior edge of S1–S4, pale brown setae; segment 1 blue; S2 blue with patch of beige at anterior end; S3 anteriorly dark blue, turning yellow posteriorly, S4–S8 yellow with patches of blue on terminal end of each segment; S9 overall brown with blue dorsal stripe extending medially lengthwise, and light patch medially in lateral view; S10 with blue patch laterally that extends to ventral surface, terminal edge raised medially to form protrusion; cerci dark brown with gold setae and appearing as a curved crescent shape dorsally, excavated medially on each lobe and sloping posteriorly; paraprocts in dorsal view dark brown and darkening apically, with bumpy texture; in the lateral perspective the lobes are roughly triangular and tapering apically to form small, acutely rounded lobes, with dorsal edge slightly uneven and ventral edge expanded to form small “hump”, terminating in small, acutely rounded lobe.

***Measurements* (mm)**: total length 38 mm, abdomen 33 mm, HW 20 mm.

###### Description of allotype.

***Head***: Labium overall pale beige; labrum blue-green, with black latero-posterior edges and a medially black spot at posterior edge; anteclypeus, genae, and mandibles (expect for reddish tips) blue-green; postclypeus darker blue with a small bar located on the medial, anterior edge; frons blue-green, abruptly changing to black posteriorly; scapes, and pedicels black, flagella dark brown and lightening apically; vertex and rear of head black with line of setae, a bronze shimmer, and white pruinescence; a pair of white post-ocular spots present; three pale ocelli with a yellow patch apical of the median ocellus; eyes green.

***Thorax***: Prothorax dorsally dark green to black with bronze shimmer; laterally blue; pronotum black medially with blue edges, latero-posterior corners rounded to obtuse angles and weakly explanate, mid-line obviously indented across the pronotum, hind lobe with raised ridge that is shorter than the width of the pronotum, is curved outward medially, and extends to a sharp point that protrudes posteriorly; mesostigmal plate black with green lateral edges, roughly triangular and not significantly raised. Pterothorax with black carina; laterally, mesepisternum with black stripe reaching the dorsal carina and reaching the mesopleural suture, but only reaching ~ 2/3 of the mesepisternum; dark green stripe located on latter 1/3 of mesepisternum, not quite reaching mesinfraepisternum, turning yellow as it passes the mesopleural suture; mesepimeron overall blue with yellow extending down from mesepisternum; dark brown line on posterior end of mesopleural suture extending ~1/6 the sutures length; metepisternum overall blue with short, black line located on metapleural suture near the base of the wings, extending ~the suture’s length; mesinfraepisternum blue; metepimeron blue; coxae, trochanters, and femora dorsally pale brown with blue patches, and ventrally pale beige with black spines; tibiae pale brown with slightly darker, and smaller, spines than that of the femora; tarsi beige with dark brown edges and small, dense spines; pale brown tarsal claws that darken apically to reddish tips, claws with a small tooth located on the basal 1/4 of their length.

***Wings***: Hyaline with dark brown venation, thickening towards the dorsal edge. Pterostigma dark brown and rhombus-shaped, with edges being the darkest; CuP slightly distal than halfway between antenodals in HW, and approximately halfway in FW; arculus originates at the second antenodal crossvein in front wings and slightly distal to it in HW; discoidal cells unequal with FW dorsal edge being 1/2 as long as HW. Nodal index: 13/2–2/11 in FW and 13/2–2/11 in HW.

***Abdomen***: Overall yellow with black dorsal stripe extending from S1–S9 and lightening laterally, with pale brown setae; S1 and S2 blue laterally; S3 blue anteriorly but turns to pale yellow posteriorly; S4–S8 pale yellow laterally; S9 with dorsal blue patch extending ¾ of its length; S10 blue dorsally and laterally. Ovipositor overall pale yellow and reddish brown ventrally, with serrated ventral edge; stylus with rounded edges, dark brown and lightening apically; gonapophysis dark reddish -brown with slightly serrated dorsal edge. Cerci roughly triangular, dark brown, and narrowing to a rounded apex, dorsal edge slightly rounded.

***Measurements* (mm)**: total length 35 mm, abdomen 28 mm, HW 20 mm.

###### Diagnosis.

**Male.***Vanuatubasisevelynae* can be distinguished from other *Vanuatubasis* species by a black pterostigma, forked (longer than wide) lateral lobes of the genital ligula, cerci curving medially their entire length, and black postclypeus with green lateral margins. **Female.***Vanuatubasisevelynae* can be distinguished from other females in this genus, by their dark colored cerci, postclypeus with dark maculation, and uneven dorsal black area on S9 appearing almost sinusoidal.

###### Variation.

**Male.** Immature specimens are pale blue with less thoracic coloring. ***Measurements* (mm)**: length 37–38 mm, abdomen 29–33 mm, HW 19–20 mm (*n* = 5). **Female.** The color of the pterostigma is lighter. ***Measurements* (mm)**: total length 35–36, abdomen 28–29 mm, HW 20–21 (*n* = 2).

###### Distribution.

Espiritu Santo, Vanuatu.

###### Etymology.

The specific epithet of this species is a Latinized noun in the genitive case of the name ‘Evelyn’ in honor of L. Evelyn Cheesman, a prominent female entomologist whose early expeditions in Vanuatu significantly paved the way for future work in this region.

###### Notes.

This species is likely the one referred to as “*Vanuatubasis* sp.” in Staniczek (2011). With the exception of one individual, all specimens were collected at the same site (Coulons, Espiritu Santo). One individuals’ label data reflects a locality on the other side of the island (Pelmol, Espiritu Santo). While this disjunct range is possible, it does seem suspect and is likely an error.

##### ﻿ Vanuatubasis insularivorum sp. nov.

Taxon classificationAnimaliaOdonataCoenagrionidae

B3A8E6E7-D545-5A55-9911-83072C3B63E2

https://zoobank.org/B70ED116-B861-42C4-AEC4-6DFC0D091B61

Figs 5 , 20C , 21D

###### Type material.

***Holotype* (1**♂ **BYU).** Male. “VANUATU: Maewo Is., | Betarara, May 23, 2019; | -15.1130, 168.0926 | Coll: SM Bybee, GS Powell | #BYU-VU-2019”.

***Paratypes* (2**♂♂ **1**♀ **BPBM, 12**♂♂ **BYU, 3**♂♂ **1**♀♀ **NHM, 4**♂♂ **1**♀♀ **NZAC). (1**♀ **BPBM, 1**♀ **BYU, 1**♂ **NHM)** same label data as holotype. **(2**♂♂ **BPBM, 2**♂♂ **BYU, 1**♂ **NHM)** “VANUATU: Maewo Is., | Betarara, May 21, 2019 | -15.1263, 168.0937 | Coll:SM Bybee,GS Powell | #BYU-VU-2019” **(3**♂♂ **BYU)** “VANUATU: Maewo Is., | Betarara, May 21, 2019 | -15.1191, 168.0891 | Coll:SM Bybee,GS Powell | #BYU-VU-2019” **(1**♂ **BYU, 1**♂ **NHM)** “VANUATU: Maewo Is., | Naone, May 24, 2019; | -15.01197, 168.0667 | Coll:SM Bybee,GS Powell | #BYU-VU-2019” **(3**♂♂ **BYU, 1**♂ **NHM)** “VANUATU: Maewo Is., | Marino, May 23, 2019; | -14.9616, 168.0605 | Coll:SM Bybee,GS Powell | #BYU-VU-2019” **(1**♂ **BYU)** “VANUATU: Maewo Is., | Baitora, May 22, 2019 | -15.1980, 168.1138 | Coll:SM Bybee,GS Powell | #BYU-VU-2019” **(1**♂ **BYU, 1**♂ **NZAC)** “VANUATU: Maewo Is., | Marino, May 23, 2019; | -14.9654, 168.0604 | Coll:SM Bybee,GS Powell | #BYU-VU-2019” **(1**♂ **NZAC)** “VANUATU: Maewo Is., | Marino, May 23, 2019; | -14.9600, 168.0614 | Coll:SM Bybee,GS Powell | #BYU-VU-2019” **(1**♀ **NZAC)** “VANUATU: Maewo Is., | Betarara, May 23, 2019 | -15.1130, 168.0926 | Coll:SM Bybee,GS Powell | #BYU-VU-2019” **(1**♂ **NZAC)** “VANUATU: Maewo Is., | Betarara, May 21, 2019 | -15.1263, 168.0937 | Coll:SM Bybee,GS Powell | #BYU-VU-2019” **(1**♂ **NZAC)** “VANUATU: Maewo Is., | Baitora, May 22, 2019 | -15.1901, 168.1107 | Coll:SM Bybee,GS Powell | #BYU-VU-2019”.

***Allotype* (1**♀ **BYU).** same label data as holotype.

###### Description of holotype.

***Head***: Labium overall pale beige; labrum blue, with dark brown latero-posterior edges and a medial brown spot at posterior edge; anteclypeus, genae, and mandibles (expect for reddish tips) blue; postclypeus blue, with a dark brown bar extending laterally, reaching the posterior edge medially, and not quite extending to anterior corners; frons greenish blue abruptly changing to black posteriorly; scapes, and pedicels greenish blue, flagella dark brown and lightening apically; vertex and rear of head dark brown; three pale ocelli with a beige patch apical of the median ocellus; eyes green.

***Thorax***: Prothorax dorsally dark brown; laterally blue; pronotum dark brown medially lightening towards the edges, latero-posterior corners rounded to obtuse angles and explanate, mid-line obviously indented across the pronotum hind lobe with raised ridge that is shorter than the width of the pronotum, appears sinusoidal with a median that extends to a sharp point that protrudes posteriorly; mesostigmal plate dark brown with blue lateral edges, roughly quadrilateral, and not raised. Pterothorax with dark brown carina; laterally, with dark brown stripe that extends laterally over the mesepisternum, reaching the mesopleural suture posteriorly across ~ 0.5 mm, but only reaching ~ 2/3 of the mesepisternum on the anterior and medial portion; pale brown stripe beginning at the mesopleural suture and extending to mesepimeron; mesepimeron overall blue with brown extending down from mesepisternum on first 1/3; metepisternum overall blue with short, dark brown line located on metapleural suture near the base of the wings, extending ~ 1/6 the suture’s length; mesinfraepisternum with dark brown spot that encompasses all but the blue posterior ventral corner; metepimeron blue and turning beige dorsally; coxae, trochanters, and femora dorsally pale brown and ventrally pale beige with dark brown spines; tibiae pale brown with slightly darker, and smaller, spines than that of the femora; tarsi beige with dark brown edges and small, dense spines; pale brown tarsal claws that darken apically to reddish tips, claws with a small tooth located on the basal 1/4 of their length.

**Figure 5.**
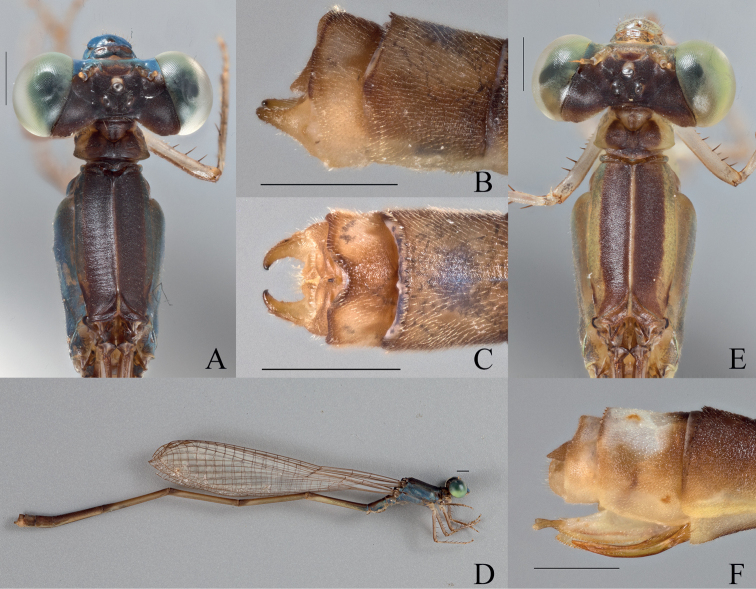
*Vanuatubasisinsularivorum* Holotype (♂ BYU) **A** dorsal thorax **B** lateral terminalia **C** dorsal terminalia **D** lateral habitus *V.insularivorum* Allotype (♀ BYU) **E** dorsal thorax **F** lateral terminalia. Scale bars: 0.5 mm.

***Wings***: Hyaline with brown venation. Pterostigma pale brown and rhombus-shaped; CuP halfway between antenodals in HW, and slightly proximal to halfway in FW; arculus originates just distal of the second antenodal crossvein in both wings; discoidal cells unequal with FW dorsal edge being 1/2 as long as HW. Nodal index: 13/2–2/12 in FW and 11/2–2/11 in HW.

***Abdomen***: Overall beige with brown dorsal stripe extending from S1–S10 and lightening laterally, brown reaching ventrally at posterior edge of S4–S6, pale brown setae; S1 and S2 blue with beige patches; S3 blue at anterior 1/3, and beige for latter 2/3; S4–S7 beige with dark brown dorsal strip reaching ventrally at posterior edge of segments; S8 brown; S9 brown with dorsal medial blue patch; S10 pale brown with darker edges. Cerci pale brown with gold setae and appearing as a curved sinusoidal shape dorsally, with abrupt ridge medially; in the lateral perspective the lobes are roughly triangular, with dorsal edge slightly sloped ventrally and appearing hooked apically. Paraprocts with dense pale brown setae that thins apically across the appendages.

***Measurements* (mm)**: total length 38 mm, abdomen 32 mm, HW 19 mm.

###### Description of allotype.

***Head***: Labium overall pale beige; labrum pale green with brown latero-posterior edges and slightly indented medially brown spot at posterior edge; anteclypeus, genae, and mandibles (expect for reddish tips) pale green; postclypeus translucent brown darker bar stretching across on the medial, anterior edge; frons pale green, abruptly changing to dark brown posteriorly, with a reddish brown spot on apex of head; scapes, and pedicels pale brown, flagella pale brown and lightening apically; vertex and rear of head dark brown with line of setae and a bronze shimmer; three pale ocelli with beige patch apical of the median ocellus; eyes pale green.

***Thorax***: Prothorax dorsally dark brown; laterally pale brown with hints of green; pronotum dark brown medially with beige edges, latero-posterior corners rounded to acute angles and weakly explanate, mid-line obviously indented across the pronotum, hind lobe with raised ridge that is shorter than the width of the pronotum, is curved outward medially, and extends to a sharp, rounded point that protrudes posteriorly; mesostigmal plate brown with green lateral edges, roughly triangular and not significantly raised. Pterothorax with dark brown carina; laterally, mesepisternum with dark brown stripe reaching the dorsal carina and reaching approximately 1/2 the mesepisternum; pale green on latter 1/3 of mesepisternum; mesepimeron overall pale green with hints of blue; dark brown line on posterior end of mesopleural suture extending ~ 1/6 the sutures length; metepisternum overall blue with short, brown line located on metapleural suture near the base of the wings, extending ~ 1/6 the suture’s length; mesinfraepisternum pale brown; metepimeron beige; coxae, trochanters, and femora dorsally pale brown and ventrally pale beige with black spines; tibiae pale brown with slightly darker, and smaller, spines than that of the femora; tarsi beige with dark brown edges and small, dense spines; pale brown tarsal claws that darken apically to reddish tips, claws with a small tooth located on the basal 1/4 of their length.

***Wings***: Hyaline with brown venation; pterostigma pale brown and rhombus-shaped; CuP halfway between antenodals in both wings; arculus at second antenodal crossvein in both wings; discoidal cells unequal with FW dorsal edge being 1/2 as long as HW. Nodal index: 15/2–2/14 in FW and 12/2–2/11 in HW.

***Abdomen***: Overall beige with brown dorsal stripe extending from S1–S8 and lightening laterally, with pale brown setae; S1 and S2 with hints of blue laterally; S3–S8 pale beige laterally, with dorsal stripe reaching ventral at terminal ends of S3–S7; S9 with pale dorsal patch extending ¾ of its length; S10 pale brown; ovipositor overall beige, with serrated ventral edge; stylus with rounded edges, pale brown and lightening apically; gonapophysis reddish brown with slightly serrated dorsal edge. Cerci roughly triangular, dark brown, and narrowing to a rounded apex, dorsal edge straight.

***Measurements* (mm)**: total length 38 mm, abdomen 31 mm, HW 23 mm.

###### Diagnosis.

**Male.***Vanuatubasisinsularivorum* can be distinguished from all other species of *Vanuatubasis* by its distinctly pointed posterior edge of the pronotum, mesostigmal plates with dorso-posterior corner not raised in an auricle, and a postclypeus with a transverse bar on the anterior edge. **Female.***Vanuatubasisinsularivorum* can be distinguished from other *Vanuatubasis* females by a postclypeus with pale maculation, a frons with dark triangle extending from base of the median ocelli, and a distinctly pointed posterior edge of the pronotum.

###### Variation.

**Male.** This species is highly variable in overall color. Some specimens are dark brown to black dorsally. Wings with dark brown venation and pterostigma. Wings with brown pigmentation. Shape of maculation on S9 somewhat variable. ***Measurements* (mm)**: total length 37–41 mm, abdomen 31–35 mm, HW 19–23 mm (*n* = 12). **Female.** Overall color varies the same as in males. ***Measurements* (mm)**: total length 38–41 mm, abdomen 31–35 mm, HW 23–24 mm (*n* = 5).

###### Distribution.

Maewo, Vanuatu.

###### Etymology.

The specific epithet of this species a combination of the Latin words *insula*, meaning island, and *rivorum* (genitive plural noun) meaning of small streams, which accurately describes the island of Maewo.

##### ﻿ Vanuatubasis kapularum sp. nov.

Taxon classificationAnimaliaOdonataCoenagrionidae

615AD87D-5D04-5B01-A7EE-47F7CE250BF9

https://zoobank.org/B7579D85-263D-4251-82AC-67AD6AD1DD4E

Fig. 6 , 20D , 21E

###### Type material.

***Holotype* (1**♂ **BYU).** “VANUATU: Efate Is., | Ulei, June 11 2019; | -17.5768, 168.2960 | Coll: SM Bybee, GS Powell | #BYU-VU-2019”.

***Paratypes* (2**♂♂ **BPBM, 7**♂♂ **BYU, 3**♂♂ **NHM, 4**♂♂ **1**♀ **NZAC). (6**♂♂ **BYU, 3**♂♂ **NHM, 2**♂♂ **NZAC).** “VANUATU: Efate Is., | Ulei, June 11 2019; | -17.5768, 168.2960 |Coll: SM Bybee, GS Powell | #BYU-VU-2019” **(1**♂ **BYU, 2**♂♂ **NZAC**) “VANUATU: Efate Is., | Mele Maat, June 11, 2019; -17.6754, 168.2559 | Coll: SM Bybee, GS Powell | #BYU-VU-2019” **(1**♀ **NZAC**) “VANUATU: Efate Is., | Devil’s Point Rd., -17.684167 |168.253638, 12.vi.2018, | coll. S. Bybee & G. Powell”.

***Allotype* (1**♀ **BYU).** “VANUATU: Efate Is., | Mele Maat, June 11, 2019; -17.6754, 168.2559 | Coll: SM Bybee, GS Powell | #BYU-VU-2019”.

###### Description of holotype.

***Head***: Labium overall pale beige; labrum blue, with black latero-posterior edges and medially, a depressed black spot at posterior edge; anteclypeus blue; genae, and mandibles (expect for reddish brown tips) blue with dark brown anterolateral edges; postclypeus overall covered by a black bar that begins medially and extends to the anterior edge, not extending to the blue, anterior corners; frons black medially, with blue lateral edges, abruptly changing to black posteriorly; scapes, and pedicels black, flagella dark brown and lightening apically; vertex and rear of head black with line of setae, a bronze shimmer, and white pruinescence; a pair of white post-ocular spots present; three pale ocelli with beige patch apical of the median ocellus; eyes green.

***Thorax***: Prothorax dorsally black with a bronze shimmer; laterally mottled appearance with dark brown, blue and hints of green; pronotum black with white pruinescence, latero-posterior corners rounded to obtuse angles and slightly explanate, mid-line obviously indented across the pronotum, hind lobe with raised ridge that is shorter than the width of the pronotum, overall sinusoidal, is curved outward medially, and extends to a sharp point that protrudes posteriorly; mesostigmal plate black with blue edges, interior edges protruding posteriorly, and raised to form very short lobes. Pterothorax with black carina; laterally, mesepisternum with black stripe reaching the dorsal carina and reaching over 1/2 the mesepimeron; blue stripe across the interpleural suture; metepisternum overall dark brown with black line located on metapleural suture near the base of the wings, extending ~ 1/6 the suture’s length; mesinfraepisternum black; metepimeron dark black and lightening anteriorly with white pruinescence; coxae, trochanters, and femora dorsally dark brown and ventrally beige with black spines; tibiae beige with smaller spines than that of the femora; tarsi beige with brown edges and small, dense spines; pale brown tarsal claws that darken apically to reddish tips, claws with a small tooth located on the basal 1/4 of their length.

**Figure 6.**
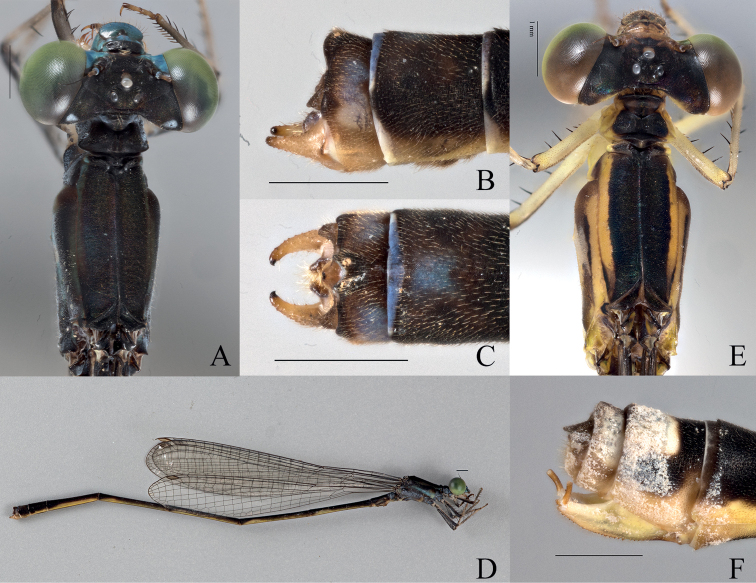
*Vanuatubasiskapularum* Holotype (♂ BYU) **A** dorsal thorax **B** lateral terminalia **C** dorsal terminalia **D** lateral habitus *V.kapularum* Allotype (♀ BYU) **E** dorsal thorax **F** lateral terminalia. Scale bars: 0.5 mm.

***Wings***: Hyaline with dark brown to black venation that thickens dorsally; pterostigma pale brown and rhombus-shaped, darkest on the edges; CuP halfway between antenodals in both wings; arculus just distal of second antenodal crossvein in both wings; discoidal cells unequal with FW dorsal edge being 1/2 as long as HW. Nodal index: 15/2–2/13 in FW and 12/2–2/12 in HW.

***Abdomen***: Overall with dark brown to black dorsal stripe extending from S1–S10 and lightening to brown laterally, brown reaching ventrally at posterior edge of S3–S6, pale brown setae; S1 blue with white pruinescence; S2–S9 yellow; S9 with dorsal blue spot on posterior end; S10 with a pair of blue patches laterally; cerci dark brown with pale brown setae and appearing as a curved crescent shape dorsally, internal margins touching continuously with black, bulbous tips; paraprocts in dorsal view dark brown and darkening apically, with bumpy texture; in the lateral perspective the lobes are roughly triangular and tapering apically to form small, acutely rounded lobe, dorsal edge sloping ventrally and ventral edge expanding ventrally to form a small lump before abruptly tapering to rounded apex.

***Measurements* (mm)**: total length 41 mm, abdomen 35 mm, HW 21 mm.

***Variation*. Male.** Blue dorsal patch on S9 variable in shape and size; S10 lateral blue spots appear cream colored in some specimens. Size of the brown stripe on the metepisternum is somewhat variable. Immature males are yellow to dark beige. ***Measurements* (mm)**: total length 39–42 mm, abdomen 33–36 mm, HW 21–22 mm (*n* = 10).

###### Description of allotype.

***Head***: Labium overall pale beige; labrum brown medially lightening towards the edges, except for dark brown postero-lateral edges and slightly indented medially dark brown spot at posterior edge; anteclypeus, genae, and mandibles (expect for reddish tips) pale brown; postclypeus translucent pale brown, with darker bar stretching across on the medial, anterior edge; frons pale brown abruptly changing to dark black posteriorly; scapes, and pedicels light brown, flagella pale brown and lightening apically; vertex and rear of head black with line of setae, a bronze shimmer, and faint white pruinescence; three pale ocelli with a beige patch apical of the median ocellus; eyes green.

***Thorax***: Prothorax dorsally black; laterally yellow with pale brown stripes; pronotum black medially with yellow lateral edges, latero-posterior corners rounded to obtuse angles and weakly explanate, mid-line obviously indented across the pronotum, hind lobe with raised ridge that is shorter than the width of the pronotum, is curved outward medially, and extends to a sharp point that protrudes posteriorly; mesostigmal black with yellow lateral edges, roughly triangular and not slightly raised medially. Pterothorax with black carina, laterally mesepisternum with black stripe reaching the dorsal carina and extending ~ 1/3 of the mesepisternum; latter 1/3 of mesepisternum yellow; mesepimeron yellow with short, black medial stripe; pale brown mesepisternum; metepisternum overall yellow, with short, black line located on metapleural suture near the base of the wings, extending ~ 1/6 the suture’s length; mesinfraepisternum yellow with dark brown medial spot; metepimeron pale yellow and lightening ventrally; coxae, trochanters, and femora dorsally brown and ventrally pale beige with black spines; tibiae pale brown with slightly darker, and smaller, spines than that of the femora; tarsi beige with dark brown edges and small, dense spines; pale brown tarsal claws that darken apically to reddish tips, claws with a small tooth located on the basal 1/4 of their length.

***Wings***: Hyaline with dark brown venation; pterostigma pale brown and rhombus-shaped; CuP halfway between antenodals in FW, and slightly proximal to halfway in HW; arculus at second antenodal crossvein in both wings; discoidal cells unequal with FW dorsal edge being 1/2 as long as HW. Nodal index: 13/2–2/12 in FW and 12/2–2/11 in HW.

***Abdomen***: Overall yellow with black dorsal stripe extending from S1–S9 and lightening towards the edges, with pale brown setae; black carina stripe extending ventrally on terminal edges of S2–S6; S9 with pale dorsal patch extending 3/4 of its length; S10 pale dorsally. Ovipositor overall beige, with brownish red serrated ventral edge, and pale brown setae; stylus with rounded edges, pale brown and lightening apically; gonapophysis reddish brown with slightly serrated dorsal edge. Cerci roughly triangular, dark brown, and narrowing to a rounded, acute point, dorsal edge straight.

***Measurements* (mm)**: total length 37 mm, abdomen 32 mm, HW 21 mm.

###### Diagnosis.

**Male.***Vanuatubasiskapularum* can be distinguished from all other *Vanuatubasis*, besides *V.nunggoli* by the presence of dark brown thoracic coloring on the metepisternum. It can be distinguished from *V.nunggoli* due to by having pale yellow paraprocts with dark tips, dorsally with inner angles forming ~ 90° angle and by the bright blue labrum. **Female.***Vanuatubasiskapularum* can be distinguished from other *Vanuatubasis* females by having a black area dorsally on S9 with relatively straight edges, dark colored cerci, and having the dorso-posterior corners of the mesostigmal plates raised in a small auricle.

###### Variation.

**Male.** Blue dorsal patch on S9 variable in shape and size; S10 lateral blue spots appear cream colored in some specimens. Size of the brown stripe on the metepisternum is somewhat variable. Immature males are yellow to dark beige. ***Measurements* (mm)**: total length 39–42 mm, abdomen 33–36 mm, HW 21–22 mm (*n* = 10). **Female.** S9 and S10 dorsal patch blue in some specimens; thoracic coloring more blue than yellow in mature specimens. ***Measurements* (mm)**: total length 37–40 mm, abdomen 32–34 mm, HW 21–22 mm (*n* = 2).

###### Distribution.

Efate, Vanuatu.

###### Etymology.

The specific epithet *kapularum* is here treated as a noun in the genitive case, in honor of the Kapula family who were among the first to show us the wonders of Vanuatu.

###### Notes.

This species was observed feeding on spiders at the Ewor River locality. One male specimen was collected with a spider in its mandibles.

##### ﻿ Vanuatubasis malekulana

Taxon classificationAnimaliaOdonataCoenagrionidae

(Kimmins, 1936)

DEB813D2-BEFC-5545-8950-0C99A38F59FF

Figs 7 , 20E , 21F

Neosbasis malekulana Kimmins, 1936: 72–73; Ober and Staniczek 2009: 492–495.

###### Remark.

*Vanuatubasismalekulana* was known by males only. Here we tentatively assign and describe the female.

###### Material examined.

***Holotype* (1**♂ **NHM).** “Holo- | type” “New Hebrides: | Malekula, | Ounua. | Mar .and Apl. 1929 | Miss L.E. Cheesman. | B.M.1929–343.” “331.” “NESOBASIS | malekulana | ♂ Holotype sp.n. | det.D.E.Kimmins.” **Additional material (2**♂♂ **BYU).** “VANUATU: Malekula Is: | Litslits, -16.1459 | 167.465, 18, 24.v.2018, | coll. S. Bybee and G. Powell”.

***Paratypes* (2**♂♂ **NHM).** “Para- | type” “New Hebrides: | Malekula, | ounua. | Feb.1929. | Miss L.E.Cheesman. | B.M.1929-234.” “231.” “NESOBASIS | malekulana | ♂ sp.n. | det.D.E.Kimmins.”.

###### Description of female.

***Head***: Labrium overall pale beige; labrum pale green with brown latero-posterior edges and a small, slightly indented medial pale spot at posterior edge; anteclypeus, genae, and mandibles (expect for reddish tips) pale green; postclypeus green with faint brown maculation medially; frons pale green, turning black posteriorly; scapes, pedicels, and flagella dark brown; vertex of head black, back of head pale green; three pale ocelli with small green patch apical of the median ocellus; eyes green.

***Thorax***: Prothorax dorsally black; laterally green; pronotum black medially with green edges, latero-posterior corners rounded to acute angles and weakly explanate, mid-line obviously indented across the pronotum, hind lobe with raised ridge that is curved outward medially, and extends to a sharp, acute point that protrudes posteriorly; mesostigmal plate black with green lateral edges, internal margins raised and protruding posteriorly. Pterothorax with black carina; laterally mesepisternum with black stripe reaching the dorsal carina and extending more than 3/4 of the mesepisternum, dark green on latter 1/4 of mesepisternum except for short brown strip at posterior edge of mesopleural suture; mesepimeron overall pale green with hints of yellow; metepisternum overall green with short, brown line located on metapleural suture near the base of the wings; mesinfraepisternum pale green to yellow; metepimeron pale green, with beige anterior portion; coxae and trochanters beige, femora dorsally dark brown and ventrally beige with black spines; tibiae pale brown with slightly darker, and smaller, spines than that of the femora; tarsi beige with dark brown edges and small, dense spines; pale brown tarsal claws that darken apically to reddish tips, claws with a small tooth located on the basal 1/4 of their length.

**Figure 7.**
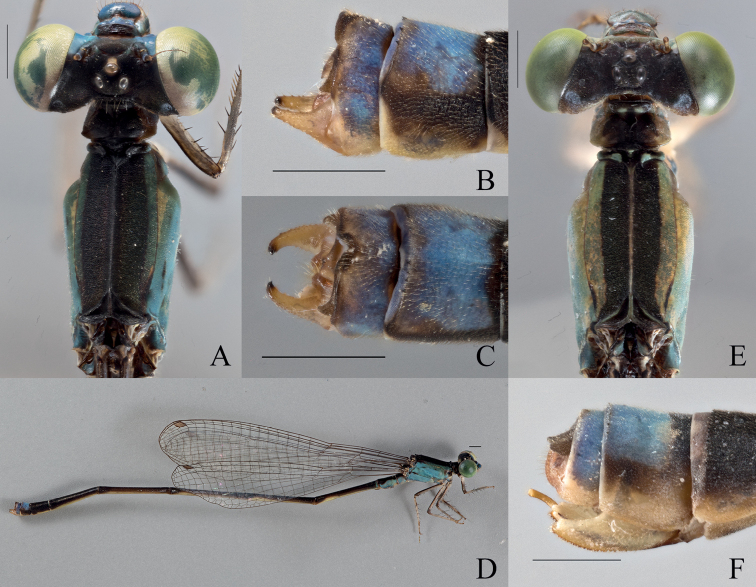
*Vanuatubasismalekulana* (♂ BYU) **A** dorsal thorax **B** lateral terminalia **C** dorsal terminalia **D** lateral habitus *V.malekulana* (♀ BYU) **E** dorsal thorax **F** lateral terminalia. Scale bars: 0.5 mm.

***Wings***: Hyaline with dark brown; pterostigma dark brown and rhombus-shaped, darkest on the edges; CuP halfway between antenodals in both wings; arculus just distal of second antenodal crossvein in both wings; discoidal cells unequal with FW dorsal edge being 1/2 as long as HW. Nodal index: 12/2–2/11 in FW and 10/2–2/9 in HW.

***Abdomen***: Overall pale green to yellow with black dorsal stripe from S1–S8 and lightening towards the edges, with pale brown setae; S1 and S2 green laterally; S3–S8 yellow laterally; S9 with pale blue dorsal stripe; S10 pale blue; Ovipositor overall beige, with darker serrated ventral edge; stylus with rounded edges, pale brown and lightening apically; gonapophysis reddish brown with slightly serrated dorsal edge. Cerci roughly triangular, brown, and narrowing to a rounded apex, dorsal and ventral edge straight.

***Measurements* (mm)**: total length 34–35 mm, abdomen 24–28 mm, HW 21–22 mm (*n* = 3).

###### Diagnosis.

**Male.***Vanuatubasismalekulana* can be distinguished from all other *Vanuatubasis*, by the dorso-posterior corner of the mesostigmal plate raised in an auricle, lack of dark color on the metepimeron. **Female.** Females of *V.malekulana* can be distinguished by the dorsal, black area on S9 having an almost straight posterior edge, and having distinctly raised dorso-posterior corners of the mesostigmal plate in an auricle.

###### Variation.

**Male.** Postclypeal maculation sometimes extending to posterior edge of postclypeus; internal projects of cerci vary from rounded to more pointed; variable extent to which internal margins of mesostigmal plates are raised; dorsal patch on S9 variable in shape and ranging in color from cream to blue. **Female.** Postclypeal maculation variable in extent; sometimes having small, brown maculation on the mesinfraepisternum.

###### Distribution.

Malekula, Vanuatu.

###### Notes.

The male of this species was recently treated in Ober and Staniczek (2009). This species is the most variable within the genus with regional differences in size and coloration that do not represent any consistent structural differences. The female is tentatively associated here as variation among the males makes it difficult to determine species limits. Future work may lead to additional new species.

##### ﻿ Vanuatubasis nunggoli sp. nov.

Taxon classificationAnimaliaOdonataCoenagrionidae

8EA4D0C0-6599-5803-BF6F-C78BE14D26C5

https://zoobank.org/89FC33DA-7362-4654-9ACD-D223677DFE50

Figs 8 , 20F , 21G

###### Type material.

***Holotype*. (1**♂ **BYU)** “VANUATU: Pentecost Is., | Wali, May 26–27, 2019; | -15.9310, 166.1897 | Coll: SM Bybee, GS Powell | #BYU-VU-2019”.

***Paratypes* (2**♂♂ **1**♀ **BPBM, 12**♂♂ **1**♀ **BYU, 3**♂♂ **1**♀ **NHM, 8**♂♂ **2**♀♀ **NZAC). (4**♂♂ **BYU, 2**♂♂ **NZAC)** “VANUATU: Pentecost Is., | Ranmawat, May 30, 2019; | -15.8126, 168.1770 | Coll:SM Bybee,GS Powell | #BYU-VU-2019” **(1**♂ **BYU, 1**♂ **NHM)** “VANUATU: Pentecost Is., | Salap, May 28, 2019; | -15.9589, 168.1948 | Coll:SM Bybee,GS Powell | #BYU-VU-2019” **(2**♂♂ **1**♀ **BPBM, 6**♂♂ **1**♀ **BYU, 2**♂♂ **1**♀ **NHM, 2**♂♂ **2**♀♀ **NZAC)** same label data as holotype. **(1**♂ **BYU, 3**♂♂ **NZAC)** “VANUATU: Pentecost Is., | Panas, May 31, 2019; | -15.9088, 168.1904 | Coll:SM Bybee,GS Powell | #BYU-VU-2019” **(1**♂ **NZAC)** “VANUATU: Pentecost Is., | St Joseph, May 31, 2019; | -15.8886, 168.1803 | Coll:SM Bybee,GS Powell | #BYU-VU-2019”.

***Allotype*** (**1**♀ **BYU).** same label data as holotype.

###### Description of holotype.

***Head***: Labium overall pale beige; labrum blue-green, with black latero-posterior edges and medially, a depressed black spot at posterior edge; anteclypeus blue-green; genae, and mandibles (expect for reddish brown tips) blue-green with darker anterolateral edges; postclypeus overall covered by a black bar that begins medially and extends to the anterior edge, not extending to the dark blue-green, anterior corners; frons black medially with blue-green lateral edges, abruptly changing to black posteriorly; scapes, and pedicels black, flagella dark brown and lightening apically; vertex and rear of head black with line of setae, a bronze shimmer, and a white pruinescence; a pair of white post-ocular spots present; three pale ocelli with small pale patch apical of the median ocellus; eyes green.

***Thorax***: Dorsally black with a bronze shimmer and white pruinescence; laterally blue and green; pronotum black, latero-posterior corners rounded to obtuse angles and explanate, mid-line obviously indented across the pronotum, hind lobe with raised ridge that is shorter than the width of the pronotum, is curved outward medially, and extends to a sharp point that protrudes posteriorly; mesostigmal plate black with blue-green lateral edges, interior edges raised dorsally to form protruding lobes. Pterothorax with black carina laterally, mesepisternum with black stripe reaching the dorsal carina and reaching the mesopleural suture posteriorly across ~0.5 mm, but only reaching ~ 2/3 of the mesepisternum on the anterior and medial portion; green stripe located on latter 1/3 of mesepisternum and extending past the dark brown mesopleural suture; mesepimeron overall blue with green extending down from mesepisternum; dark brown stripe extending from mesinfraepisternum to ~ 0.25 mm from the base of the wings; metepisternum overall blue with short, brown line located on metapleural suture near the base of the wings, extending ~ 1/6 the suture’s length; mesinfraepisternum dark brown; metepimeron dark brown on dorsal 1/2, and blue ventrally, with white pruinescence; coxae, trochanters, and femora dorsally dark brown and ventrally pale beige with brown spines; tibiae pale brown with smaller spines than that of the femora; tarsi beige with dark brown edges and small, dense spines; pale brown tarsal claws that darken apically to reddish tips, claws with a small tooth located on the basal 1/4 of their length.

**Figure 8.**
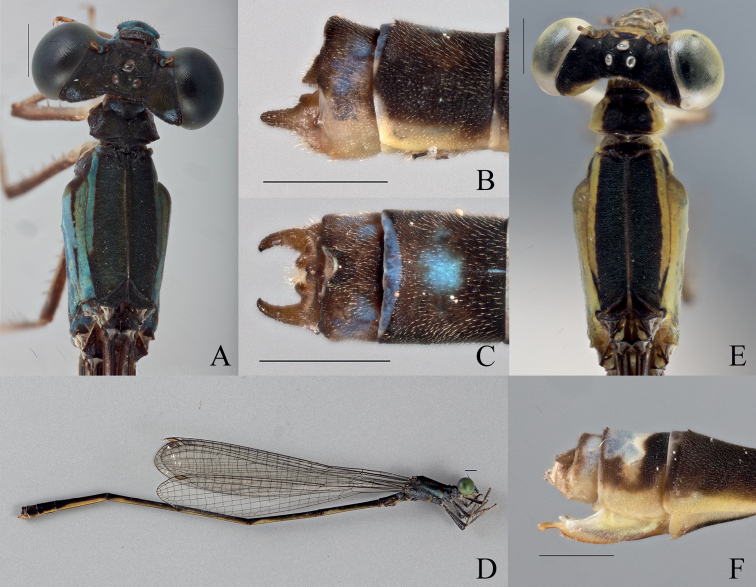
*Vanuatubasisnunggoli* Holotype (♂ BYU) **A** dorsal thorax **B** lateral terminalia **C** dorsal terminalia **D** lateral habitus *V.nunggoli* Allotype (♀ BYU) **E** dorsal thorax **F** lateral terminalia. Scale bars: 0.5 mm.

***Wings***: Hyaline with dark brown venation; pterostigma pale brown and rhombus-shaped, darkest on the edges; CuP slightly proximal to halfway between antenodals in both wings; arculus just distal of second antenodal crossvein in both wings; discoidal cells unequal with FW dorsal edge being 1/2 as long as HW. Nodal index: 14/2–2/13 in FW and 12/2–2/11 in HW.

***Abdomen***: Overall yellowish with black dorsal stripe extending from S1–S9 and lightening to brown laterally, brown reaching ventrally at posterior edge of segments 2–5, with pale brown setae; S1 and S2 mottled blue and brown; S3 anteriorly dark blue, turning yellow posteriorly, S4–S8 yellow; S9 overall brown with blue dorsal stripe medially; S10 with blue lateral spots, terminal edge raised medially to form protrusion. cerci dark brown with pale brown setae and appearing as a curved crescent shape dorsally, medial edges touching at base and apex, terminating in darkened round lobes; paraprocts in dorsal view dark brown and darkening apically, with bumpy texture, slightly converging medially continuously that form sharp terminal hooks; in the lateral prospective the cerci are roughly triangular and tapering apically, with dorsal edge slightly uneven and ventral edge slightly expanded, terminating in small, acutely rounded lobe.

***Measurements* (mm)**: total length 42 mm, abdomen 35 mm, HW 21 mm.

###### Description of allotype.

***Head***: Labium overall pale beige; labrum pale blue with brown latero-posterior edges and a slightly indented medially brown spot at posterior edge; anteclypeus, genae, and mandibles (expect for reddish tips) pale blue; postclypeus translucent brown; frons yellow, abruptly changing to black posteriorly; scapes, and pedicels pale blue, flagella pale brown and lightening apically; vertex and rear of head black with line of setae and a bronze shimmer; three pale ocelli with small beige patch apical of the median ocellus; eyes pale green.

***Thorax***: Dorsally black; laterally yellow with hints of blue; pronotum black medially with yellow lateral edges, latero-posterior corners rounded to obtuse angles and weakly explanate, mid-line obviously indented across the pronotum, hind lobe with raised ridge that is shorter than the width of the pronotum, is curved outward medially, and extends to a sharp, rounded point that protrudes posteriorly; mesostigmal plate brown with yellow lateral edges, not significantly raised dorsally. Pterothorax with black carina; laterally, mesepisternum with black stripe reaching the dorsal carina and extending ~ 1/2 of the mesepisternum, reaching the mesopleural suture posteriorly across ~ 0.5 mm, but only reaching ~ 1/2 of the mesepisternum on the anterior and medial portion; pale green on ventral 1/2 of mesepisternum with hint of a brown stripe; metepisternum overall yellow with short, brown line located on metapleural suture near the base of the wings, extending ~ 1/6 the suture’s length; mesepimeron overall pale blue; dark brown line on posterior end of mesopleural suture extending ~ 1/6 the sutures length; mesinfraepisternum pale yellow with apical brown spot; metepimeron light yellow; coxae and trochanters beige, femora dorsally pale brown and ventrally pale beige with black spines; tibiae pale brown with slightly darker, and smaller, spines than that of the femora; tarsi beige with dark brown edges and small, dense spines; pale brown tarsal claws that darken apically to reddish tips, claws with a small tooth located on the basal 1/4 of their length.

***Wings***: Hyaline with dark brown to black venation that thickens dorsally; pterostigma pale brown and rhombus-shaped, darkest on the edges; CuP halfway between antenodals in both wings; arculus just distal of second antenodal crossvein in both wings; discoidal cells unequal with FW dorsal edge being 1/2 as long as HW. Nodal index: 15/2–2/13 in FW and 12/2–2/12 in HW.

***Abdomen***: Overall yellow with black dorsal stripe extending from S1–S8 and lightening towards the edges, with pale brown setae; S1 and S2 with hints of blue and green laterally; S3 apically blue and turning yellow posteriorly; S3–S7 pale yellow laterally, with dorsal stripe reaching ventral at terminal ends; S8 yellow; S9 with pale dorsal patch extending ¾ of its length; S10 pale brown with pale dorsal patch; ovipositor overall beige, with darker serrated ventral edge; stylus with rounded edges, pale brown and lightening apically; gonapophysis reddish brown with slightly serrated dorsal edge. Cerci roughly triangular, dark brown, and narrowing to a rounded apex, dorsal and ventral edge straight.

***Measurements* (mm)**: total length 38 mm, abdomen 32 mm, HW 21 mm.

###### Diagnosis.

**Male.***Vanuatubasisnunggoli* can be distinguished from all other *Vanuatubasis*, besides *V.kapularum* by the presence of dark brown thoracic coloring on the metepisternum. It can be distinguished from *V.kapularum* by the inner posterior angle of the paraprocts forming an obtuse angle and the green-blue labrum. **Female.***Vanuatubasisnunggoli* can be distinguished from other females in this genus, by the dorsal black area on S9 with an uneven posterior edge (this coloring on S9 is sharper than that in *V.evelynae* and not extending as far), a short pedicel, a brown pterostigma, and a blueish hue on the pterothorax.

###### Variation.

**Male.** The males of this species exhibit variable thoracic coloring, with some males almost black and others blue-green with only traces of black striping. This variation may be connected to populations as males from certain localities seemed to have more dark thoracic coloring than others. ***Measurements* (mm)**: total length 39–42 mm, abdomen 34–37 mm, HW 21–23 mm (*n* = 14). **Female. *Measurements* (mm)**: total length 37–40 mm, abdomen 31–33 mm, HW 21–24 mm (*n* = 6).

###### Distribution.

Pentecost, Vanuatu.

###### Etymology.

The specific epithet of this species is a noun in apposition, derived from the local word for “land diving,” a cultural practice that originated on the island of Pentecost.

##### ﻿ Vanuatubasis rhomboides sp. nov.

Taxon classificationAnimaliaOdonataCoenagrionidae

183685F2-C9E7-523D-AE56-04B1C8CB424C

https://zoobank.org/3B882901-4835-4CC1-ADFA-B9EE493D41AD

Figs 9 , 21H

###### Type material.

***Holotype* (1**♀ **NZAC).** (NZAC04230978, New Zealand Arthropod Collection, Auckland, New Zealand), locality data: Republic of Vanuatu, Malekula Island, Stretch of Lakatchkach River flowing through Postanle Area (-16.1437, 167.4671 to -16.1474, 167.4649; 15–51 m a.s.l.): 17 May 2017; M. Marinov & S. Bybee leg.

***Paratype* (1**♀ **NZAC).** NZAC04231074, Malekula Island, Litslits (-16.1435, 167.4671; 15 m a.s.l.): 07 May 2019, S. Bybee & G. Powell leg.

###### Description of holotype.

***Head***: Labium pale yellow, labrum, mandibles (except for the reddish tips), whole clypeus, frons and genae along the eyes up to the dorsal ends of the scapes, scapes citron yellow except for dark fuscous to dark reddish spots at the dorsal corners of the mandibles at the ends of the lateral proximal corners of the postclypeus plus lateral sides of labrum running down for almost the whole length to the anterior edge; pedicels brownish; flagella dark red; scape: pedicel 0.5; vertex black with slight dark red sheen with three yellow spots – two are horn-like expansions of the yellow on the postfrons into the dark vertex and the third is just in front of the median ocellus; rear part of the head yellow which is continuing up toward the occipital area and visible from the dorsum in a shape of two pear-like yellow areas on the postocular lobes and a small spot on the occipital bar; two roughly circular occipital spots formed by pale pruinescence; eyes orange yellow with pale fulvous areas on the dorsal part.

***Thorax***: Dark wide bar (slightly tapering towards posterior end) with slight reddish sheen passing on the dorsal part of pro- and pterothorax encompassing ~ 3/4 of the dorsal part of the mesostigmal plate and 1/2 of mesepisternum (faint pale line along the dorsal carina starting approximately midway and gradually becoming lighter towards the pre-alar area), at the posterior end the dark bar abruptly curves ventrally at a near 90° angle and joins the posterior end of mesopleural suture with a semicircular curvature; dark elongated spots with same color developed also as follows: two sitting on the dorsal edges of the meso- and metapleural sutures at their posterior ends and connecting to two dark shiny spots at the alar areas by a tiny bridge with the same color and a solid bar on the posterior edge of poststernum; legs almost entirely yellow save for darken areas on the inner surfaces of the front tibia in the proximal 2/3 and dorsal part of all femora occupying as follows: distal 1/2 of front femora, 9/10 of the distal part in the middle and hind legs, all dark areas with characteristic pale spots giving a barred-like appearance, pale spots on front femora weakly developed forming serrated outer edges; dark spots at the joints between femora and tibiae and distal tips of the last tarsal segments; claws orange with darker reddish tips; all the leg spines black; mesostigmal plate triangular in shape with dorsoposterior end enlarged and extruded into an auricle-like lobe, ventral side wide rounded and slightly elevated and separated from the thorax; hind lobe of the prothorax is triangular with two parallel carinae ventrally arising out from the lateral sides of the hind lobe and running posteriorly to the dorsal edge.

***Wings***: Hyaline; venation generally dark especially at the distal ends becoming paler towards the bases with pale spots at the nodus at both outer and inner sides; pterostigma with protruding posterior proximal end into a sharp angle which is dissimilar in both front and hind wings differing in the following: front wing smaller with central fuscous area outlined with pale yellow, hind wing larger with anterior end 2/3 of the length of posterior, bicolored with dark area continuing towards the sharp proximal end of the pterostigma and pale yellow anterior part; CuP slightly proximal to halfway between antenodals in front wings and halfway in hind wings sitting on the top CuP and AA distal at the point where the latter is leaving the wing edge; arculus at second antenodal in front wings and slightly distal to it in hind wings; discoidal cells dissimilar in shape – in front wings anterior side is ~ 1/3 of the posterior and in hind wings anterior side is more than ½ of posterior; two postdiscoidal cells before nodus; nodal index: 12/2–2/12 in front wings and 10/2–2/10; MA and MP very long reaching to surpassing the midway between nodus and pterostigmas, CuA surpassing the nodus and ends proximal to the midway between nodus and pterostigma.

***Abdomen***: Black dorsal area running from S1 to S8 continuing on the dorsal 1/2 of S9 where it descends on the lateral side of the segment outlining a wide circular beige spot on each side of the segment and joining the posterior end of the segment, dark area on the dorsum of S9 ends up at two round lobes, posterior dorsal 1/2 of S9 with a pale brownish bar, S10 with a dark area on the dorsum which is constricted at both lateral sides by the beige expansions from the lateral sides of the segment giving the dark area a roughly X-shape when viewed from the dorsum; dark area with dorsal invaginations forming lateral bulbous expansions at proximal ends on S2 and S3 and yellow bars continuing up almost meeting on the dorsum of S3–S5; ventral side of the abdomen yellow with slim dark lines on the ridges of the sternites 2–8, those lines faint on second segment occupying approximately mid-area and continuing more towards the anterior part of the segment, slightly skewed towards the anterior end on segments 3–5 and equidistant from both ends of the segments on 6–8; posterior end of the eight sternite lacking tooth; cerci fuscous with pale tips; ovipositor yellow surpassing the posterior end of S10 with the tip reaching just before the distal tips of the cerci, styli yellow at the extreme base and gradually darkening becoming deep dark red distally with very weak yellow lightening at the tips, styli surpassing the cerci.

**Figure 9.**
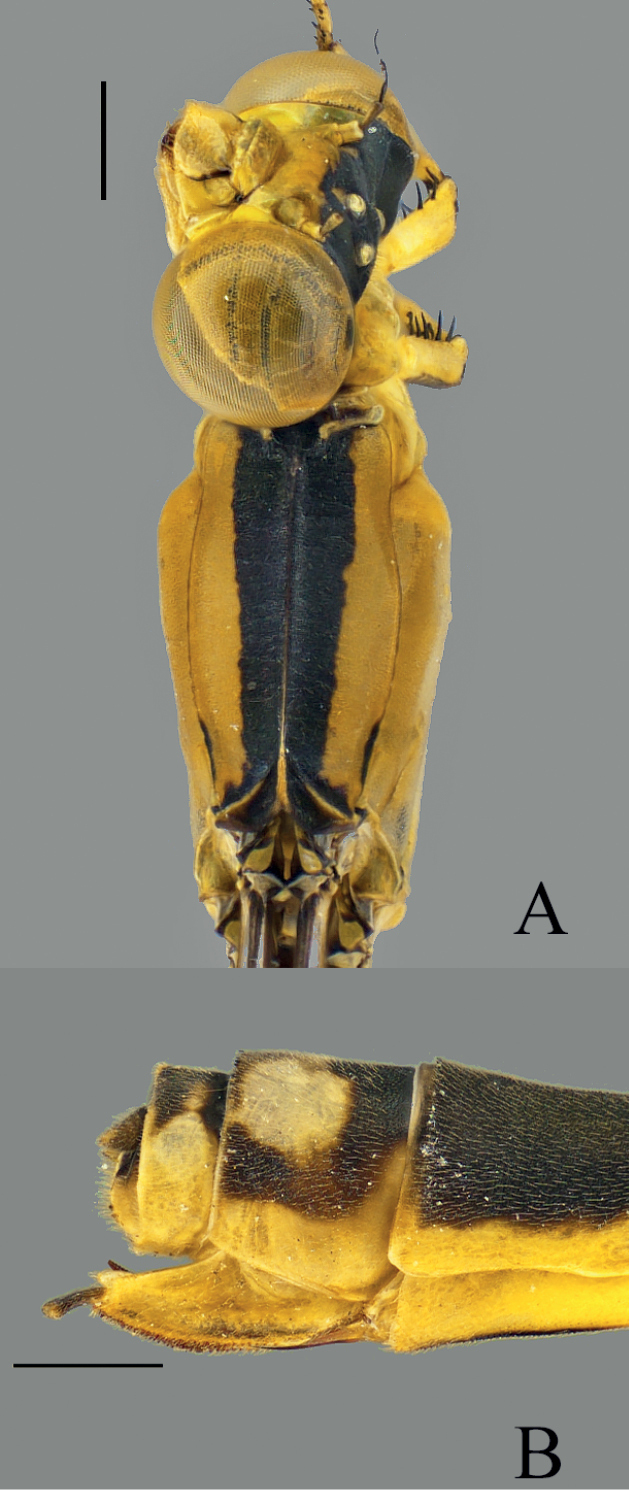
*Vanuatubasisrhomboides* Holotype (♀ NZAC) **A** dorsal thorax **B** lateral terminalia. Scale bars: 0.5 mm.

***Measurements* (in mm)**: total length 35.5, abdomen 30.0, hind wing 22.5.

###### Diagnosis.

*Vanuatubasisrhomboides* has a mesostigmal plate with the dorso-posterior end raised as an auricle. This shape is found only in three other congeners: *V.kapularum*, *V.nunggoli*, and *V.malekulana.* The height of the auricle is less pronounced in the first two species, but *V.malekulana* has a similarly high auricle to *V.rhomboides*. The latter is distinguished by all other congeners by the rhomboidal shape of the pterostigmata which are characteristically bicolored in HW.

###### Variation.

Body overall green and predominantly yellow on the ventral side; dark area on the dorsum of S9–10 reduced. ***Measurements*** (in mm): total length, abdomen 30.0, hind wing 22.5.

###### Distribution.

Malekula, Vanuatu.

###### Etymology.

The name *rhomboides* is Latinized form of Greek *ῥομβοειδής*, –*ής*, –*έϛ* = like a rhombus, rhomboid, in reference to the shape of the pterostigmata {adjective}.

###### Notes.

This species is only known from female specimens as no males were able to be associated.

##### ﻿ Vanuatubasis santoensis

Taxon classificationAnimaliaOdonataCoenagrionidae

Ober & Staniczek, 2009

D8738B6A-CDCB-5BC9-AC8E-9FE4849ED810

Figs 10 , 20G , 21I

Vanuatubasis santoensis Ober & Staniczek (2009): 487–490.

###### Remark.

*Vanuatubasissantoensis* was known by males only. Here we describe the female.

###### Material examined.

***Holotype* (1**♂ **SMNS).** “Vanuatubasis santoensis ♂ HOLOTYPE | Ober and Staniczek 2009 | Vanuatu, Sanma Province | Espiritu Santo, surroundings of | Penaoru, Penaoru River|leg. A.H. Staniczek and M. Pallmann, 13.XI.2006 | det. S.V. Ober, 21.VII.2008 | coll.- | Ober and Staniczek 2009 | 14.96105°S, 166.63316°E 90 m | ODO 000242 K.” **(4**♂♂ **BYU)** “VANUATU: Santo Is: | Wailapa, -15.5781 | 167.0024, 6.vi.2018, coll, | S. Bybee and G. Powell” **(1**♂ **BYU)** “VANUATU: Santo Is: | Narango, -15.6274 | 166.8535, 4.vi.2018, | coll, S. Bybee and G. Powell” **(2**♂♂ **BYU)** “VANUATU: Santo Is: | Narango, -15.5538 | 166.9814, 4.vi.2018, | coll, S. Bybee and G. Powell” **(12**♂♂ **4**♀♀ **BYU)** “VANUATU: Santo Is: | Ipayato, -15.6296| 166.8426, 4 vi.2018. | coll. S. Bybee and G. Powell” **(5**♀♀ **BYU)** “VANUATU: Santo Is: Felea | -15.3839 166.8426, 4.vi.2018, | coll, S. Bybee and G. Powell”

***Paratypes* (2**♂♂ **SMNS).** same label data as Holotype with the following barcode numbers: ODO 000246 K, ODO 000247 K. **(1**♂ **SMNS).** “Vanuatu, Sanma Province | Espiritu Santo, surroundings of Tasmate, Paé River, 15.2175°S, 166.68706°E, 139 m, 11.XI.2006, leg. A.H. Staniczek and M. Pallmann”, with the following barcode number: ODO 000245 K. **(2**♂♂ **SNMS, 2**♂♂ **MNHN).** “Vanuatu, Sanma Province, Espiritu Santo, surroundings of Tasmate, Mamasa River, 15.20976°S, 166.67705°E, 20 m, 9.XI.2006, leg. A. H. Staniczek and M. Pallmann”, SNMS specimens with the following barcodes: ODO 000243 K, ODO 000244 K.

###### Description of the female.

***Head***: Labium overall pale beige; labrum pale green with brown latero-posterior edges and small, slightly indented medially brown spot at posterior edge; anteclypeus, genae, and mandibles (expect for reddish tips) pale green; postclypeus translucent brown with green lateral edges; frons pale green, turning black posteriorly; scapes, pedicels, and flagella pale brown; vertex and rear of head black with line of setae and a bronze shimmer; three pale ocelli with small beige patch apical of the median ocellus; two white post-ocular spots; eyes pale green.

***Thorax***: Prothorax dorsally black; laterally green; pronotum black medially with brown edges, latero-posterior corners rounded to acute angles and weakly explanate, mid-line obviously indented across the pronotum, hind lobe with raised ridge that is shorter than the width of the pronotum, is curved outward medially, and extends to a sharp, rounded point that protrudes posteriorly; mesostigmal plate black with green lateral edges and not significantly raised. Pterothorax with black carina; laterally mesepisternum with black stripe reaching the dorsal carina and extending more than 3/4 of the mesepisternum, pale green on latter 1/4 of mesepisternum; mesepimeron overall pale green with hints of yellow; metepisternum overall green with short, brown line located on metapleural suture near the base of the wings, extending ~ 1/6 the suture’s length; mesinfraepisternum pale green with apical brown spot; metepimeron green, with beige anterior portion; coxae and trochanters beige, femora dorsally dark brown and ventrally beige with black spines; tibiae pale brown with slightly darker, and smaller, spines than that of the femora; tarsi beige with dark brown edges and small, dense spines; pale brown tarsal claws that darken apically to reddish tips, claws with a small tooth located on the basal 1/4 of their length.

**Figure 10.**
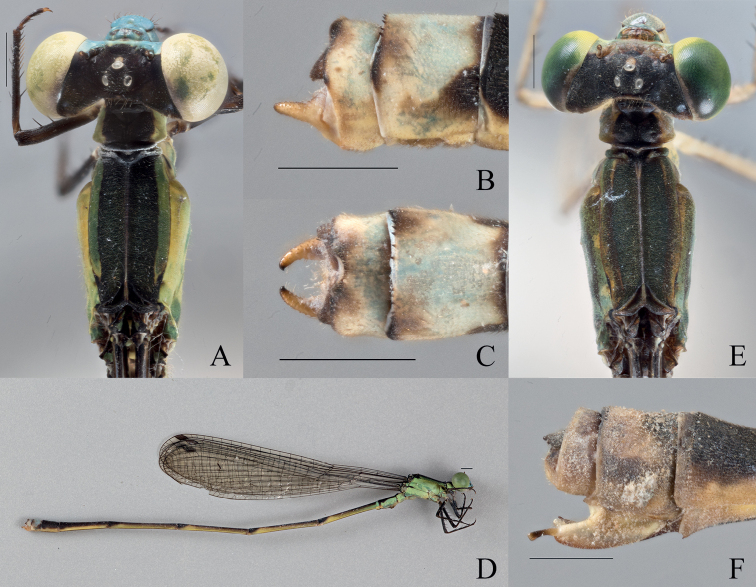
*Vanuatubasissantoensis* (♂ BYU) **A** dorsal thorax **B** lateral terminalia **C** dorsal terminalia **D** lateral habitus *V.santoensis* (♀ BYU) **E** dorsal thorax **F** lateral terminalia. Scale bars: 0.5 mm.

***Wings***: Hyaline with dark brown; pterostigma dark brown and rhombus-shaped, darkest on the edges; CuP halfway between antenodals in both wings; arculus just distal of second antenodal crossvein in both wings; discoidal cells unequal with FW dorsal edge being 1/2 as long as HW. Nodal index: 13/2–2/12 in FW and 12/2–2/11 in HW.

***Abdomen***: Overall beige yellow with black dorsal stripe from S1–8 and lightening towards the edges, with pale brown setae; S1 and S2 green laterally; S3–S8 yellow laterally; S9 with pale dorsal stripe; S10 pale brown; ovipositor overall beige, with darker serrated ventral edge; stylus with rounded edges, pale brown and lightening apically; gonapophysis reddish brown with slightly serrated dorsal edge. Cerci roughly triangular, dark brown, and narrowing to a rounded apex, dorsal edge straight a ventral edge slightly rounded ventrally.

***Measurements* (mm)**: total length 36–39 mm, abdomen 29–33 mm, HW 22–24 mm (*n* = 9).

###### Diagnosis.

**Male.***Vanuatubasissantoensis* can be distinguished from other *Vanuatubasis* by its black pterostigma, pale blue postclypeus without any bars or maculations, dorso-posterior corner of mesostigmal plate not raised in an auricle (or if elevated, only slightly so). **Female.***Vanuatubasissantoensis* can be distinguished from other females in this genus, by its dark brown to black pterostigma, uniformly colored postclypeus (sometimes with pale spots), frons with a dark triangle extending from the base of the median ocelli, and the dark area on the mesepisternum reaching the black spot at the posterior end of the mesopleural suture.

###### Variation.

**Male.** Postclypeal maculation more prominent than in some individuals. Pterothorax sometimes with variable short black stripes. **Female.** Thoracic coloring varies from yellow to green, presumably due to the age of the individual.

###### Distribution.

Espiritu Santo, Vanuatu.

##### ﻿ Vanuatubasis xanthochroa sp. nov.

Taxon classificationAnimaliaOdonataCoenagrionidae

9BDE3563-583A-51F1-A885-FF851E0409A8

https://zoobank.org/3F3CD171-A876-49FE-93EA-D69D512A288B

Figs 11 , 21J

###### Type material.

***Holotype* (1**♀ **NZAC**). “Republic Of Vanuatu, Malekula Island, | Stretch of Lakatchkach River flowing through Postanie Area | 16.1437S, 167.4671E; 15 m a.s.l. | 17 May 2017 | M. Marinov & S. Bybee leg.” “*Vanuatubasis* sp.2., female | M. Marinov det., August 2017” “NZ Arthropod Collection | Private Bag 92170 | Auckland | New Zealand | NZAC04230976”.

***Paratypes* (2**♀♀ **BPBM, 5**♀♀ **BYU, 3**♀♀ **NHM, 4**♀♀ **NZAC). (2**♀♀ **BPBM, 4**♀♀ **BYU, 3**♀♀ **NHM, 3**♀♀ **NZAC**) “VANUATU: Malekula Is: | Litslits, -16.14594705 | 167.4653247, 18, 24.v.2018 | coll. S. Bybee and G. Powell.” **(1**♀ **BYU, 1**♀ **NZAC)** “VANUATU: Malekula Is., | Litslits, May 7^th^ 2019 | -116.1435, 167.4671 | Coll:SM Bybee, GS Powell | VU-BYU-2019”.

###### Description of holotype.

***Head***: Labium pale yellow; labrum, mandibles (except for the reddish tips), whole clypeus, frons and genae along the eyes up to the dorsal ends of the scapes, scapes and pedicels citron yellow except for a dull fulvous (to pale brownish) spot at the middle of the labrum along its posterior edge with triangular shape and two fuscous spots at the postero-lateral corners of the labrum; flagella dark red; scape: pedicel 0.5; vertex black with slight dark red sheen with three yellow spots – two are expansions of the yellow face into the dark vertex and the third is just in front of the median ocellus; rear part of the head yellow which is continuing up toward the occipital area and visible from the dorsum on the posterior ends of postocular lobes and occipital bar; two roughly circular occipital spots formed by pale pruinescence; eyes orange yellow with pale fulvous areas on the dorsal part; eyes with three transverse lines in right eye and one in left which are unclear if are post mortem or present in life.

***Thorax***: Entire thorax including the legs yellow with pale fulvous area starting from the dorsal part of the thorax and diffusing around the level of mesepimeron; five dark spots as follows: two faint sitting on the dorsal end of mesopleural and metapleural sutures (metapleural very obscure) almost touching the posterior corners of both sutures; two dark red at the dorsal posterior corners of both mesepimeron and metepimeron and one at the posterior end of the poststernum; leg spines deep dark red to almost black, claws fulvous becoming darker at the tips; mesostigmal plate, roughly quadrilateral-shaped with expanded dorsoanterior side thus wider than ventral side; hind lobe of the prothorax raised, roughly triangular shape with two parallel carinae, dorsal angulated and ventral rounded arising out from the lateral sides of the hind lobe, running posteriorly to the dorsal edge.

**Figure 11.**
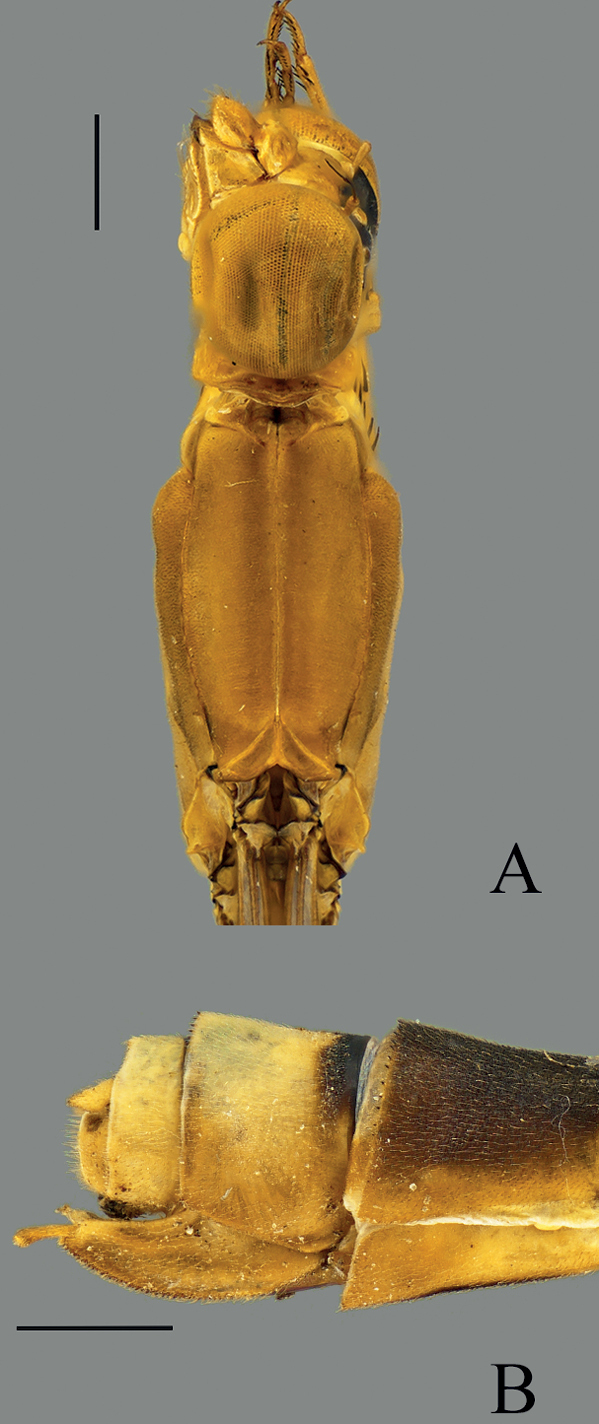
*Vanuatubasisxanthochroa* Holotype (♀ NZAC) **A** dorsal thorax **B** lateral terminalia. Scale bars: 0.5 mm.

***Wings***: Hyaline; venation generally dark especially at the distal ends becoming paler towards the bases with pale spots at the nodus at the outer sides; pterostigma rhomboidal fulvous with pale yellow lines along the edges that are wider on the dorsal edge and faint to almost not existing at the anterior edge; CuP halfway between antenodals in front wings and closer to second antenodal in hind wings situated proximally to the wing petiolation and distant from the point where CuP and AA is leaving the wing edge for nearly a whole of its length; arculus distal from the second antenodal in all wings; discoidal cells dissimilar in shape – in front wings anterior side is ~ 1/4 of the posterior and in hind wings anterior side is ~ 1/2 of posterior; three postdiscoidal cells before nodus; nodal index: 12/2–2/11 in front wings and 10/2–2/11; MA, MP and CuA very long reaching to surpassing the midway between nodus and pterostigmas.

***Abdomen***: Generally dark fulvous on the dorsum and pale yellowish on the ventral side with the following peculiarities: fulvous dorsal area is very faint to almost missing on S1 and gradually becoming darker towards the posterior end finishing abruptly at ~ 1/4 of S9, remainder of S9 and S10 pale cream with a touch of a faint blue on the dorsum, dorsum of S10 at the intersegmental membrane to S9 with a very narrow dark red bar not continuing on the lateral sides of the segment; dorsum of S2–S7 with anterior part paler, becoming darker at the posterior ~ 1/6–1/7 end of the segments, all with yellow bars at the anterior end continuing from the venter and almost touching on the dorsum, S8 uniformly dark; small tooth at the posterior end of the eight sternite; cerci pale yellow; ovipositor orange yellow surpassing the posterior end of S10 with the tip aligned with the tips of cerci and styles surpassing the cerci.

***Measurements* (in mm)**: total length 33.5, abdomen 28.0, hind wing 20.5.

###### Diagnosis.

**Female.***Vanuatubasisxanthochroa* can be distinguished from all other *Vanuatubasis* females by the lack of a black dorsal stripe across the carina and no postclypeal maculation.

###### Variation.

**Female.** Dark spot on the labrum larger; both eyes with transverse lines; posterior end of the posterior edge of the prothorax triangular shape and not as wide as in the holotype; CuP situated at the petiolation at the base of CuP and AA where the later leaves the wing edge, pterostigma with yellow lines all around the edges, nodal index: 11/2–2/11 in front wings and 9/2–2/9 in hind wings, spine on the ventral side of eight sternite large and sharp.

###### Distribution.

Malekula, Vanuatu

###### Etymology.

The name *xanthochroa* is Latinized feminine form of Greek *ξανθόχρους*, –*ους*, –*ουν* = yellow colored, in reference to the color of the thorax {declinable adjective}.

###### Notes.

This species is only known from female specimens as no males were able to be associated.

### ﻿Key to species of *Vanuatubasis* using mature males

**Table:** 

1	Terminal segment of genital ligula with lobes laterally covering the sclerotized portion of the first segment (Fig. 12A), or lobes extended in parallel sided, longer than wide, projections (Fig. 12C)	**2**
–	Terminal segment of genital ligula with lobes laterally not covering the sclerotized portion of the first segment (Fig. 12B); lobes not extended in parallel sided projections (Fig. 12D)	**3**
2	Paraprocts subparallel, only curving medially at apical 1/3. Cerci with two acute teeth. Aneityum Is.	***V.bidens***
–	Paraprocts strongly curving medially their entire length. Cerci lacking two teeth. Espiritu Santo Is.	***V.evelynae***
3	Mesostigmal plate with dorso-posterior corner not raised in an auricle; if elevated then height of the projection is less than 1/2 the distance between the tip of the auricle and the dorsal carina (Fig. 13A)	**4**
–	Mesostigmal plate with dorso-posterior corner raised in an auricle; the height of the projection is greater than or equal to 1/2 the distance between the tip of the auricle and the dorsal carina (Fig. 13B)	**5**
4	Postclypeus pale blue (sometimes with yellow dots) but lacking dark markings (Fig. 14A). Espiritu Santo Is.	***V.santoensis***
–	Postclypeus pale blue with black transverse bar at the anterior edge (Fig. 14B); Maewo Is.	***V.insularivorum***
5	Mes- and metinfraepisterna almost entirely black (Fig. 15A)	**6**
–	Mes- and metinfraepisterna almost entirely pale saved for a black dot on the anterior corner of mesinfraepisternum (Fig. 15B); Malekula Is.	***V.malekulana***
6	Cerci dorsally having the inner posterior angle form a slightly acute to quadrate (~90°) angle (Fig. 16A); Efate Is.	***V.kapularum***
–	Cerci dorsally having the inner posterior angle form a broadly rounded, obtuse angle (Fig. 16B); Pentecost Is.	***V.nunggoli***

**Figure 12.**
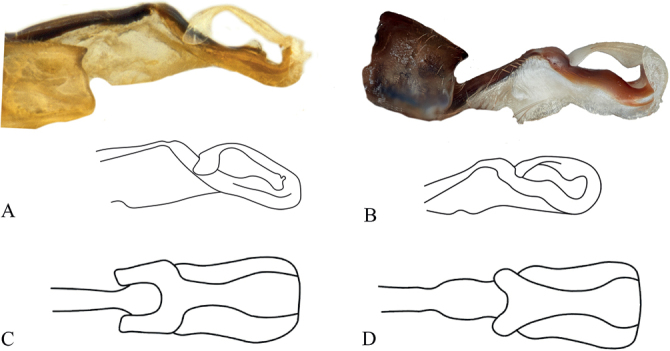
Characters of the genital ligula **A***V.bidens* image and line drawing of lateral lobes covering the sclerotized portion of the first segment **B***V.kapularum* image and line drawing of lateral lobes not covering the sclerotized portion of the first segment **C***V.evelynae* dorsal view of lateral lobes being longer than wide **D***V.malekulana* dorsal view of lateral lobes not being longer than wide.

**Figure 13.**
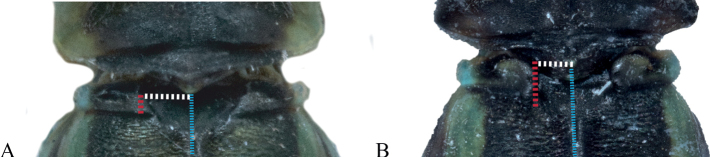
Dorsal view of mesostigmal plates showing the relative heights of the auricles **A***V.santoensis*, height < distance between tip and the dorsal carina **B***V.nunggoli*, height > distance between tip and the dorsal carina. The red dotted line represents the height of the mesostigmal plate, the white dotted line shows the distance from the the tip of the plate to the dorsal carina, and the blue dotted line runs along the dorsal carina.

**Figure 14.**
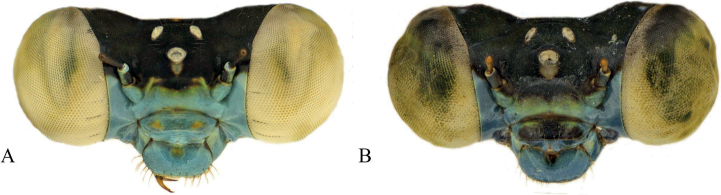
Face of male **A***V.santoensis***B***V.insularivorum*.

**Figure 15.**
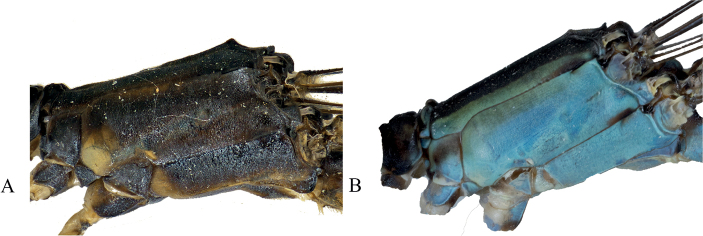
Mes- and metinfraepisterna **A***V.kapularum***B***V.malekulana*.

**Figure 16.**
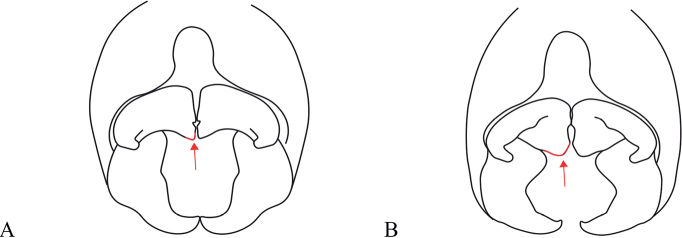
Paraprocts with inner posterior angle highlighted **A***V.kapularum* angle slightly acute to 90° **B***V.nunggoli* angle obtuse.

### ﻿Key to species of *Vanuatubasis* using mature females

**Table:** 

1	Postclypeus uniformly colored (Fig. 21H, J) OR with pale spots (Fig. 21A, D, I)	**2**
–	Posclypeus not uniformly colored; spots or darker area brown or black (Fig. 21B, C, E, F, G)	**6**
2	Cerci brown; frons various colors but always with dark semicircle on apical 1/3 (Fig. 17A)	**3**
–	Cerci pale yellow; frons yellow and lacking dark semicircle on apical 1/3 (Fig. 17B)	**5**
3	Pterostigmata of HW unicolored (Fig. 18A)	**4**
–	Pterostigmata of HW bicolored (Fig. 18B); Malekula Is.	***V.rhomboides***
4	Wing venation pale brown; pterostigmata pale yellow; black on the mesepisterna does not cover the dorsal carina; sternum of S8 without spine above the ovipositor (Fig. 5E, F); Maewo Is.	***V.insularivorum***
–	Wing venation black; pterostigmata dark; black on the mesepisterna partly cover the dorsal carina; sternum of S8 with spine above the ovipositor (Fig. 10E, F); Espiritu Santo Is.	***V.santoensis***
5	Pterothorax unicolored; Malekula Is.	***V.xanthochroa***
–	Pterothorax not unicolored; Aneityum Is.	***bidens***
6	Black area on the disk of S9 with uneven posterior edge (Fig. 21B, C, G, H)	**7**
–	Black area on the disk S9 with almost straight posterior (Fig. 21E, F)	**9**
7	Cerci brown	**8**
–	Cerci black. Espiritu Santo Is.	***V.evelynae***
8	Pedicel long (scape: pedicel < 0.4); dark area (if present) on mesinfraepisternum with a maximum width of ~1/6 of the disk; pterothorax with no blueish hue; Malekula Is.	***V.discontinua***
–	Pedicel short (scape: pedicel ≥ 0.4); dark area on mesinfraepisternum with width ≥ 1/4 of the disk; pterothorax with blueish hue; Pentecost Is.	***V.nunggoli***
9	Height of the dorso-posterior corners of mesostigmal plates equal or longer than the distance between the tip of the plate and dorsal carina (Fig. 19A); Malekula Is.	***V.malekulana***
–	Height of the dorso-posterior corners of mesostigmal plates shorter than the distance between the tip of the plate and dorsal carina (Fig. 19B); Efate Is	***V.kapularum***

**Figure 17.**
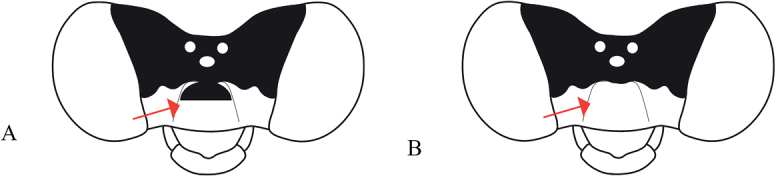
Head of females **A** with a dark semicircle on frons **B** without a dark semicircle on frons.

**Figure 18.**
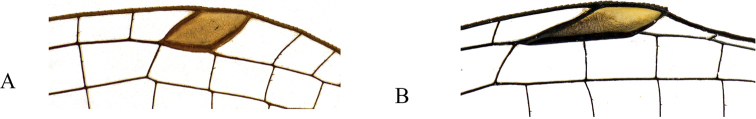
Female pterostigma **A***V.insularivorum* unicolored **B***V.rhomboides* bicolored.

**Figure 19.**
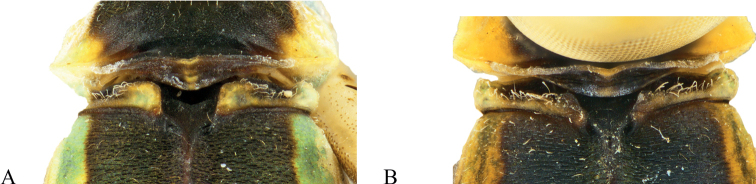
Female mesostigmal plates **A***V.malekulana***B***V.kapularum*.

## ﻿Discussion

*Vanuatubasis* exhibits great levels of island endemism, a pattern also seen in other endemic damselfly genera on Pacific island systems such as *Nesobasis* in Fiji (Donnelly 1990) and *Megalagrion* in Hawaii (Polhemus 1997). *Vanuatubasis* in overall appearance and structure most closely resembles *Nesobasis*, with the most obvious difference being the length of the cerci compared to the paraprocts. *Vanuatubasis* species, in contrast to those of *Nesobasis*, do not display a wide diversity of color to the human eye. Almost all specimens collected (excluding teneral individuals) are generally dark blue and green hues, with few exceptions (e.g., a few females of *Vanuatubasis* are yellow). In *Nesobasis* the color variation includes bright red or yellow males, as well as both pale and dark blue species.

**Figure 20.**
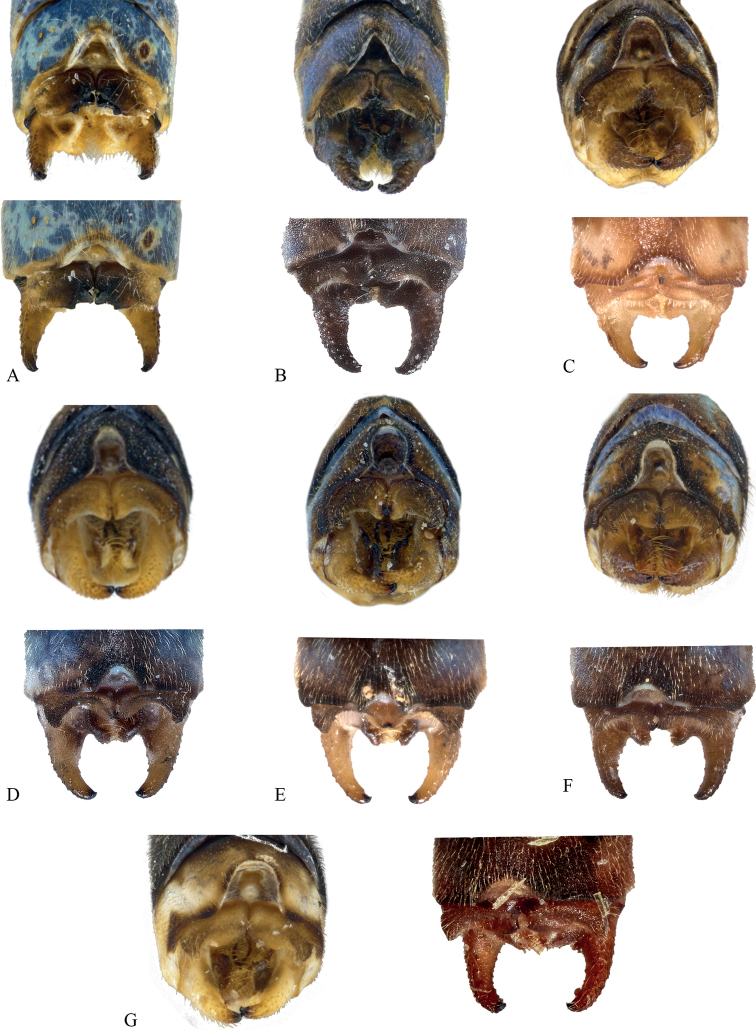
Male dorso-posterior and dorsal terminalia of **A***V.bidens***B***V.evelynae***C***V.insularivorum***D***V.kapularum***E***V.malekulana***F***V.nunggoli***G***V.santoensis*.

**Figure 21.**
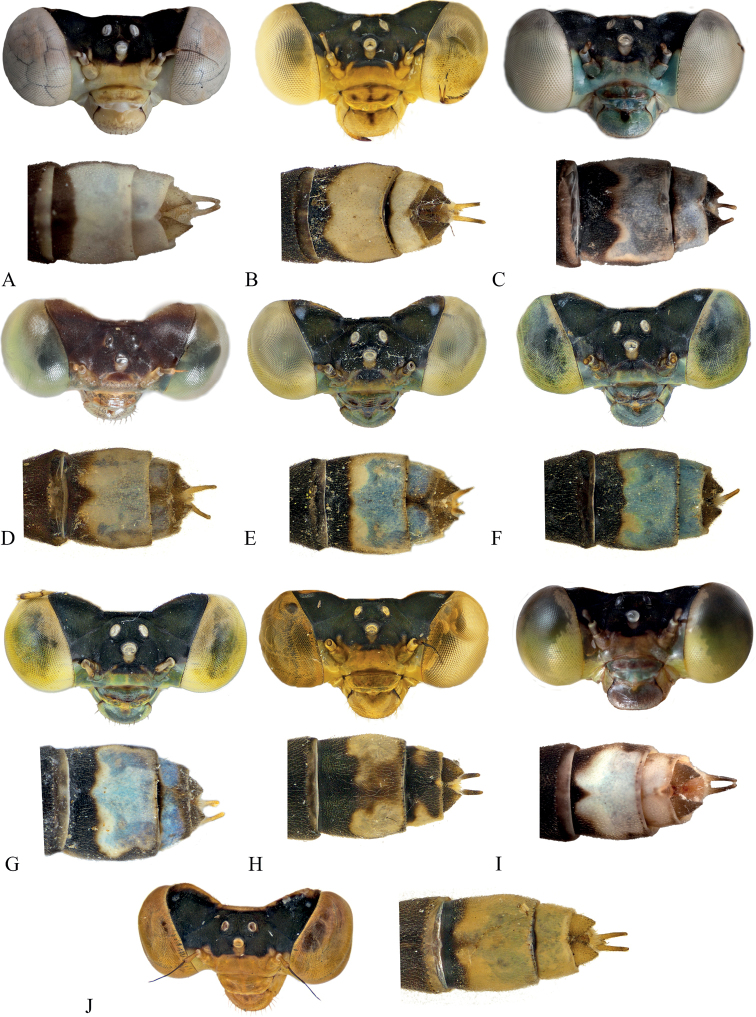
Female face and dorsal terminalia of **A***V.bidens***B***V.discontinua***C***V.evelynae***D***V.insularivorum***E***V.kapularum***F***V.malekulana***G***V.nunggoli***H***V.rhomboides***I***V.santoensis***J***V.xanthochroa*.

It appears likely that small stretches of ocean between islands provide some sort of barrier between populations allowing for speciation events to occur. This is illustrated by each species only being found on a single island in the archipelago. The high level of endemism that these species have puts them at risk due to the rapid environmental change currently taking place in Vanuatu. Over the course of several years of fieldwork, the authors noted alteration of stream habitats, such as increased agriculture and removal of native vegetation. In one instance, we noted a difference in the actual presence of some of these species on particular streams. For example, in 2018 specimens of *V.kapularum* were collected on Ewor River in Efate. The following year at the same time of year no specimens were found on the same river, and there was an overall difference in the amount of the native vegetation, with many ferns being replaced by agricultural crops. This revision looked over material collected on six islands. Continued sampling is particularly pressing, especially on additional islands, as much of the preferred habitat for the genus is quickly being altered (Wairiu 2017; Saxton et al. 2021).

*Vanuatubasis* diversity further demonstrates the need for more odonate research in the South Pacific region. This research lays the groundwork for future research on their ecology, biogeography, and evolutionary history. Work in this region should further focus on the relationships among endemic genera in Fiji and Vanuatu (e.g., *Nesobasis*, *Melanesobasis*, *Vanuatubasis*) and their placement within the larger subfamilies of Coenagrionidae. A broader understanding of their phylogenetic position will help to answer questions relating to their biogeography.

## Supplementary Material

XML Treatment for Vanuatubasis

XML Treatment for Vanuatubasis bidens

XML Treatment for Vanuatubasis discontinua

XML Treatment for Vanuatubasis evelynae

XML Treatment for Vanuatubasis insularivorum

XML Treatment for Vanuatubasis kapularum

XML Treatment for Vanuatubasis malekulana

XML Treatment for Vanuatubasis nunggoli

XML Treatment for Vanuatubasis rhomboides

XML Treatment for Vanuatubasis santoensis

XML Treatment for Vanuatubasis xanthochroa
